# The structure-function analysis of the Mpr1 metalloprotease determinants of activity during migration of fungal cells across the blood-brain barrier

**DOI:** 10.1371/journal.pone.0203020

**Published:** 2018-08-30

**Authors:** Sarisa Na Pombejra, Mantana Jamklang, John P. Uhrig, Kiem Vu, Angie Gelli

**Affiliations:** Department of Pharmacology, School of Medicine, University of California, Davis, California, United States of America; University of Minnesota, UNITED STATES

## Abstract

Cryptococcal meningoencephalitis, the most common form of cryptococcosis, is caused by the opportunistic fungal pathogen, *Cryptococcus neoformans*. Molecular strategies used by *C*. *neoformans* to invade the central nervous system (CNS) have been the focus of several studies. Recently, the role of a novel secreted metalloprotease (Mpr1) in the pathogenicity of *C*. *neoformans* was confirmed by studies demonstrating that Mpr1 mediated the migration of fungal cells into the CNS. Given this central function, the aim here was to identify the molecular determinants of Mpr1 activity and resolve their role in the migration of cryptococci across the blood-brain barrier (BBB). The Mpr1 protein belongs to an understudied group of metalloproteases of the M36 class of fungalysins unique to fungi. They are generally synthesized as propeptides with fairly long prodomains and highly conserved regions within their catalytic core. Through structure-function analysis of Mpr1, our study identified the prodomain cleavage sites of Mpr1 and demonstrated that when mutated, the prodomain appears to remain attached to the catalytic C-terminus of Mpr1 rendering a nonfunctional Mpr1 protein and an inability for cryptococci to cross the BBB. We found that proteolytic activity of Mpr1 was dependent on the coordination of zinc with two histidine residues in the active site of Mpr1, since amino acid substitutions in the HExxH motif abolished Mpr1 proteolytic activity and prevented the migration of cryptococci across the BBB. A phylogenetic analysis of Mpr1 revealed a distinct pattern likely reflecting the neurotropic nature of *C*. *neoformans* and the specific function of Mpr1 in breaching the BBB. This study contributes to a deeper understanding of the molecular regulation of Mpr1 activity and may lead to the development of specific inhibitors that could be used to restrict fungal penetration of the CNS and thus prevent cryptococcal meningoencephalitis-related deaths.

## Introduction

*Cryptococcus neoformans* is a fungal pathogen that primarily affects individuals with a compromised immune system [1, 2]. Inhalation of spores/yeast cells leads to dissemination of *C*. *neoformans* from the lung, the primary site of infection, to the brain. Once in the brain, development of fungal meningoencephalitis causes high morbidity and accounts for 15% of AIDS-related deaths worldwide [3, 4]. Movement across the blood-brain barrier (BBB) is highly restricted in order to protect the central nervous system (CNS), but cryptococci can invade the CNS by breaching brain capillary endothelial cells, which constitute the BBB.[5] Several studies have demonstrated that freely moving cryptococci in the bloodstream can cross the BBB via a transcellular pathway through brain capillary endothelial cells and also cause disruption and remodeling of tight junction proteins suggesting a paracellular path.[6–10] In addition, studies have demonstrated that cryptococci also make use of a stealthy mechanism that involves penetrating the BBB by hiding within monocytes, referred to as the Trojan horse mechanism [11–14].

Over the past few years several studies have identified fungal gene products that contribute to fungal dissemination to the CNS.[7, 9, 15–17] In an effort to address the notion that the extracellular proteome of *C*. *neoformans* was the basis for the association of cryptococci with the brain endothelium, we identified an uncharacterized, secreted metalloprotease (Mpr1) belonging to the M36 peptidase family, referred to as fungalysins.[18, 19] The M36 enzymes are unique to fungi and their multiple sequence alignment has revealed a high degree of conservation in the active site regions of the catalytic core.[20]

Some members of the M36 class function as mediators of fungal pathogenesis; for example MEP2 and MEP3 of *Microsporum canis*, and MEP4 and MEP5 of *Trichophyton mentagrophytes* promote cutaneous infections [21–23]. The M36 archetype enzyme in *Aspergillus fumigatus* hydrolyzes elastin suggesting a likely role in the fungal colonization of the lung.[24]. In the case of Mpr1, its role in the pathogenicity of *C*. *neoformans* was confirmed by both in vitro and in vivo studies demonstrating that Mpr1 promoted the migration of fungal cells into the CNS [7, 25]. The in vivo studies of both inhalation and tail-vain mouse models revealed a significant improvement in survival rates and less fungal-burden in brains of mice infected with the a strain of *C*. *neoformans* lacking Mpr1.[7] Furthermore, Mpr1 appeared to target the brain specifically, since Mpr1 was not required for fungal colonization of other organs [7, 25]. The central role of Mpr1 in breaching the BBB was further demonstrated by the acquired ability of *S*. *cerevisiae* to cross the BBB upon the sole expression of the Mpr1 cDNA from *C*. *neoformans*.[7] The aim of this study was to perform a structure-function analysis of the Mpr1 determinants of activity given its central role in the migration of *C*. *neoformans* across the BBB.

## Materials and methods

### In silico bioinformatic analysis

Protein searches and domain identification were analyzed using UniProt database (http://www.uniprot.org/). The amino acid comparison of Mpr1 was performed by ClustalW2 (http://www.ch.embnet.org/-software/ClustalW.html) and MUSCLE (http://www.ebi.ac.uk/Tools/msa/muscle/). For the tree construction, sequences with shared homology with Mpr1 were obtained by searching the HMMER database, using the hmmsearch algorithm (hmmer.janelia.org). Thirty-five sequences were chosen from the search results to represent a wide variety of, human pathogens, plant pathogens, and non-pathogens where the E-values were below 10^−100^. Peptide sequences were aligned using ClustalW, and trees were constructed using MrBayes, using 100,000 tree generations with a sampling frequency of 10, with MCMC parameters of 2 runs and 4 chains. A consensus tree and bootstrap values were produced within MrBayes using the 50 percent majority rule method.

### Mutations and expression of Mpr1 in *C*. *neoformans* and *S*. *cerevisiae*

The wild type *C*. *neoformans* var. *grubii* strain used in the study was H99 and the metalloprotease Mpr1 deletion strain (*mpr1Δ*) was previously constructed from the H99 background.[7] We constructed two cryptococcal reconstituted strains tagged with 6X-HIS (histidine) and MYC sequences at the C-termini. The cDNA of *MPR1* tagged with either 6X-HIS or MYC was cloned into pCR^TM^2.1-TOPO vector (TOPO TA Cloning Kit, K4520; Invitrogen Life Technologies, Carlsbad, CA, USA) under the endogenous promoter and terminator of Mpr1 and transformed into the *mpr1Δ* deletion strain together with pJMM180 (URA5 for selective marker) via biolistic transformation. This created the *MPR1*^*WT*^ strain.

To create a strain of *Saccharomyces cerevisiae* expressing CnMPR1, the cDNA plus 6X-HIS at the C-terminus was inserted into plasmid p416 (ATCC# 87360; American Type Culture Collection, Manassas, VA, USA), an episomal yeast shuttle vector, under the constitutive GPD (glyceraldehyde 3-phospate dehydrogenase) promoter and CYC-1 (cytochrome c-1) terminator and transformed into the wild type strain of *S*. *cerevisiae* (W303) using electroporation.[7] It should be noted that *MPR1* or M36-related metalloproteases are not normally present in the genome of *Saccharomyces cerevisiae*.[7]

For the mutations of the first histidine and the glutamic acid within HExxH, ApaI restriction site was introduced into the sequence encoding the histidine and the glutamic acid by the forward primer 5’ GTAGGGCCCCAGCCACGGTCTCTCAACTCG 3’ and the reverse primer 5’ TACGGGCCCTGAATGACAATACCAGCCTCG 3’ resulting in a change of the amino acid residues from HEYSH to QGPSH. The second histidine substitution used in MPR1^QGA^ and MPR1^QGAAA^ was made from primer design and PCR techniques to substitute the last histidine to an alanine codon (forward primer: 5’ GTAGGGCCCCAGCGCCGGTCTCTCAACTCG 3’, reverse primer: 5’ TACGGGCCCTGAATGACAATACCAGCCTCG 3’). The EGWGD mutation to AGWGA of MPR1^QGAAA^ was performed using the Site-Directed Mutagenesis Kit (Phusion Site-Directed Mutagenesis Kit, F541; Thermo Fisher Scientific) with the specific primer designs, the forward primer 5’ GGCGCTGCTATTGCCAC 3’ and the reverse primer 5’ GGTATGGGTGCGGGATGG 3’. This technique was also used to mutate D^353^ and D^355^ to alanines in MPR1^ProMut^ (forward primer: 5’ ACTCAGGGATAAAATTGAGATGATGAAGG 3’, reverse primer: 5’ TCATCAATGGCAAAGGCACTCGC 3’).

Strains of *S*. *cerevisiae* expressing mutant CnMPR1 were obtained from the introduction of episomal p416 plasmids carrying CnMPR1^QG^, CnMPR1^QGA^, or CnMPR1^QGAAA^ into the wild type strain (W303) by chemical transformation. The transformation of *MPR1* mutants into the *C*. *neoformans mpr1Δ* deletion strain was done via TOPO vectors containing the desired *MPR1* mutants and co-transformed with pJmm180 (URA5 marker) by biolistic transformation. This approach resulted in the *MPR1*^*QG*^, *MPR1*^*QGA*^, and *MPR1*^*QGAAA*^ strains.

### Southern blot and real time PCR analysis of biolistic transformants

Mpr1 proteolytic and Mpr1 prodomain mutants were constructed using biolistic transformation according to standard methods.[26] Transformants were analyzed for *MPR1* genomic copy number via either Southern blot or real time PCR. Southern blot was done using Digoxigenin labeled probes and standard protocols were followed.[27] Briefly, gDNA was isolated from wild-type, Mpr1 proteolytic and Mpr1 prodomain mutants, digested with AatII overnight, fractionated on agarose gel, transferred to a Nylon+ membrane, and probed with Digoxigenin labeled DNA probes. The following primers were used for Southern blot analysis, Mpr1_cDNA_517F: CAC AAG GCC CAC TTG GCT GAG; Mpr1_cDNA_1414R: CAT TGG TAG GTC GGT AGT TGA GCA. Gene dosage (i.e. copy number) of *MPR1* in Mpr1^QGA^ and Mpr1 ^QGAAA^ proteolytic mutant strains were also determined using real time PCR according to the method of Shepherd et al.[28] Two different sets of primers for *MPR1* were used to examine gene copy number: First set: Mpr1_RT_F1: AGC CCG CCT TTG CTG AAC and Mpr1_RT_R1: CAA CGC CAG CCT TGG AAA. Second set of primers: Mpr1_RT_F2: ACA CCC CAC CAC ACT CTC AAG and Mpr1_RT_R2: GCG CCA GAA ATG ACC TCA GT. Cch1, a high affinity calcium channel, is present as a single copy and is used as a single-copy gene control. The primers used for *CCH1* are: Cch1_RT_F1: GCG GTA CCA CCA CCA TTC TT and Cch1_RT_R1: GAA TAT GCA CGC AGC AAA AAC A.

### Yeast cell cultures and growth curves

All strains of *C*. *neoformans* were cultured in YPD (yeast peptone dextrose) media. The wild type and the mutant strains of *S*. *cerevisiae* were cultured in synthetic defined (SD) and uracil dropout media, respectively. For the growth curve, at 0 hour each cryptococcal strain with an optical density of 0.1 (OD^600^ = 0.1) was grown in YPD in an orbital shaker at 37°C. The absorbance of each cryptococcal culture was measured hourly at OD_600_ and plotted (Spectronic GENESYS 6; Thermo Electron Corp., Waltham, MA, USA).

### Association and transcytosis assay

An in vitro model of the BBB was used for association and transcytosis assays as previously described.[7, 29–31] In this model, the hCMEC/D3 cell line (human brain capillary microvascular endothelial cells [29, 31, 32] obtained from B. Weksler, Cornell University) between passages 20 and 30 was grown on collagen-coated transwells (Collagen I, Rat Tail; Corning, Discovery Labware Inc., Bedford, MA, USA; Cell Culture Insert PET membrane 8.0 μm pore size; Corning Inc., Lowell, MA, USA) submerged in the cell culture media (EGM-2 BulletKit and EBM-2 Basal Medium; Lonza, Walkerville, MD, USA) at 37°C and 5% CO_2_. The media was changed every three to four days to a 1/2 dilution and to two 1/4 dilutions respectively in order to reduce growth factors and to allow the cells to differentiate.[7, 29, 30]

Approximately 10^6^ yeast cells were added into each transwell and incubated at 37°C, 5% CO_2_. For the transcytosis assays, following co-incubation time of 6 h or 18 h, the contents of the bottom transwell was collected, placed onto to YPD agar plates and incubated at 30°C for 48 h in order to determine CFUs. For the association assays, following 1 h post-incubation, hCMEC/D3 were extensively washed with PBS. Subsequently, water was added to burst the brain endothelial cells, and the liquid was collected and plated on YPD agar plates. The data were presented in percent migration and percent association that normalized the infection numbers and the numbers of yeasts passing through and attaching to the BBB respectively. Statistical significance was determined by un-paired t-test with Welch’s correction (GraphPad-Prism5).

To monitor BBB integrity, transepithelial electrical resistance (TEER) was consistently monitored by an endometer (World Precision Instruments, Sarasota, FL, USA). Additionally, FITC-dextran permeability (Fluorescein Isothiocyanate-dextran mol wt 70,000; Sigma-Aldrich, Saint Louis, MO, USA) was performed in parallel to association and transcytosis assays by adding 1 μg/μl final concentration of FITC- conjugated dextran into the transwells (upper chambers) and subsequently quantifying the fluorescence intensity of the media in the upper and the lower chambers at 538 nm (excitation of 485 nm) using a microplate reader (SpectraMax M5; Molecular Devices, Sunnyvale, CA, USA) and SoftMax Pro 5.2 software. [31]

### Isolation of recombinant CnMPR1 and protease activity assay

*S*. *cerevisiae* was used as an expression system to produce recombinant CnMPR1 for both the wild type and the mutant proteins. The recombinant proteins, which contained 6X-HIS at the C-terminus, were purified using immobilized affinity chromatography (IMAC). The yeast were grown in SD media overnight, collected, and lysed in IMAC-Extract buffer (50mM Tris-HCl pH 7.5, 150mM NaCl, 5mM Imidazole, 5mM Dithiothreitol, 1% Nonident P-40) supplemented with protease inhibitors (complete ULTRA Tablets, Mini, EDTA-free EASYpack; Roche, Mannheim Germany). The lysates were centrifuged at 25,000xg for 1 h to collect clear supernatants. Supernatants were added to Roche complete Ni-NTA beads (c0mplete His-Tag Purification Resin; Roche, Mannheim Germany) and incubated at 4°C overnight on a nutator. Subsequently the supernatant and bead solutions were washed three times with IMAC-Wash I (50mM Tris-HCl pH 7.5, 150mM NaCl, 5mM Imidazole, 5mM Dithiothreitol) and one time with IMAC-Wash II (50mM Tris-HCl pH 7.5, 150mM NaCl, 7.5mM Imidazole, 5mM Dithothreitol) then eluted with IMAC-Elute (50mM Tris-HCl pH 7.5, 150mM NaCl, 200mM Imidazole, 5mM Dithiothreitol). The eluted proteins were concentrated using Amicon filters (Amicon Ultra-0.5 ml centrifugal filter 30K; EMD Milipore Corp., Darmstadt, Germany).

The protease assays were performed according to the manufacturer’s protocol (Fluorescent Protease Assay Kit 23266, Pierce Biotechnology, Rockford, IL, USA). Briefly, fluorescein-labeled casein was used as a substrate and a change of fluorescence properties by enzyme digestion was measured using fluorescent reader (SpectraMax M5; Molecular Devices, Sunnyvale, CA, USA; SoftMax Pro 5.2 software) at 485/538nm (excitation/emission). The recombinant CnMPR1^WT^ and CnMPR1^QGAAA^ purified by IMAC were tested for their proteolytic activity. Trypsin was used as a positive control for the assay.

### Western blot analysis

The yeast cells were lysed with Lysis Buffer (50mM Tris-HCl pH 7.5, 150mM NaCl, 5mM NaF, 5mM EDTA, 0.1% NP-40) supplemented with 1mM DTT and yeast protease inhibitors (Protease Inhibitor Cocktail P8215; Sigma-Aldrich, Saint Louis, MO, USA). The protein concentrations were quantified using Bradford assay (Quick Start^TM^ Bradford Protein Assay; Bio-Rad Laboratories Inc., Hercules, CA, USA). Then the samples were denatured and reduced in Laemmli buffer (Premixed 4X Laemmli Protein Sample Buffer; Bio-Rad Laboratories Inc., Hercules, CA, USA) and boiled at 95°C for 3 minutes. Electrophoresis was performed using 10% SDS-PAGE gel at 80V for 3.5 hours (Mini-PROTEAN Tetra Cell; Bio-Rad Laboratories Inc., Hercules, CA, USA). The proteins on SDS-PAGE gel were transferred to a polyvinylidene difluoride membrane (Immun-Blot PVDF membrane; Bio-Rad Laboratories Inc., Hercules, CA, USA) using semi-dry transfer method (Trans-Blot SD Semi- Dry Transfer Cell; Bio-Rad Laboratories Inc., Hercules, CA, USA) at 15V for 20 minutes. The PVDF membrane was stained with Ponceau Red (Ponceau S Stain; AMRESCO LLC, Solon, OH, USA) to visualize the proteins followed by blocking with 5% milk (Blotting-Grade Blocker; Bio-Rad Laboratories Inc., Hercules, CA, USA) for 30 minutes and subsequently incubated with 1:1000 dilution of the primary antibody (Rabbit Anti-Myc tag Antibody ab9106; Abcam Inc., Cambridge, MA, USA) at 4°C overnight. The membrane was washed three times with TBST buffer and incubated with 1:5000 dilution of the secondary antibody (Goat Anti-Rabbit IgG H&L HRP ab6721; Abcam Inc., Cambridge, MA, USA) at room temperature for 1 h. After washing with TBST, the chemiluminescent substrate solution for HRP (SuperSignal West Pico Chemiluminescent Substrate; Pierce Biotechnology, Rockford, IL, USA) was added to the membrane, and the X-ray films (CL-X Posure Film; Pierce Biotechnology, Rockford, IL, USA) were used to detect the presence of the protein. For the protein loading control, the PVDF membranes were stripped and re-probed with 1:1000 dilution of GAPDH (glyceraldehyde 3-phosphate dehydrogenase) (Mouse Anti-GAPDH antibody ab125247; Abcam Inc., Cambridge, MA, USA) according to the Abcam protocol followed by using the Goat Anti-Mouse IgG HRP as the secondary antibody (ab6789; Abcam Inc., Cambridge, MA, USA).

### Statistical analysis

Statistical significance was determined by un-paired t-test with Welch’s correction (GraphPad-Prism7). P values < 0.05 were considered significant.

## Results

### Mpr1 belongs to the M36 class of fungalysins

**Fig 1:**
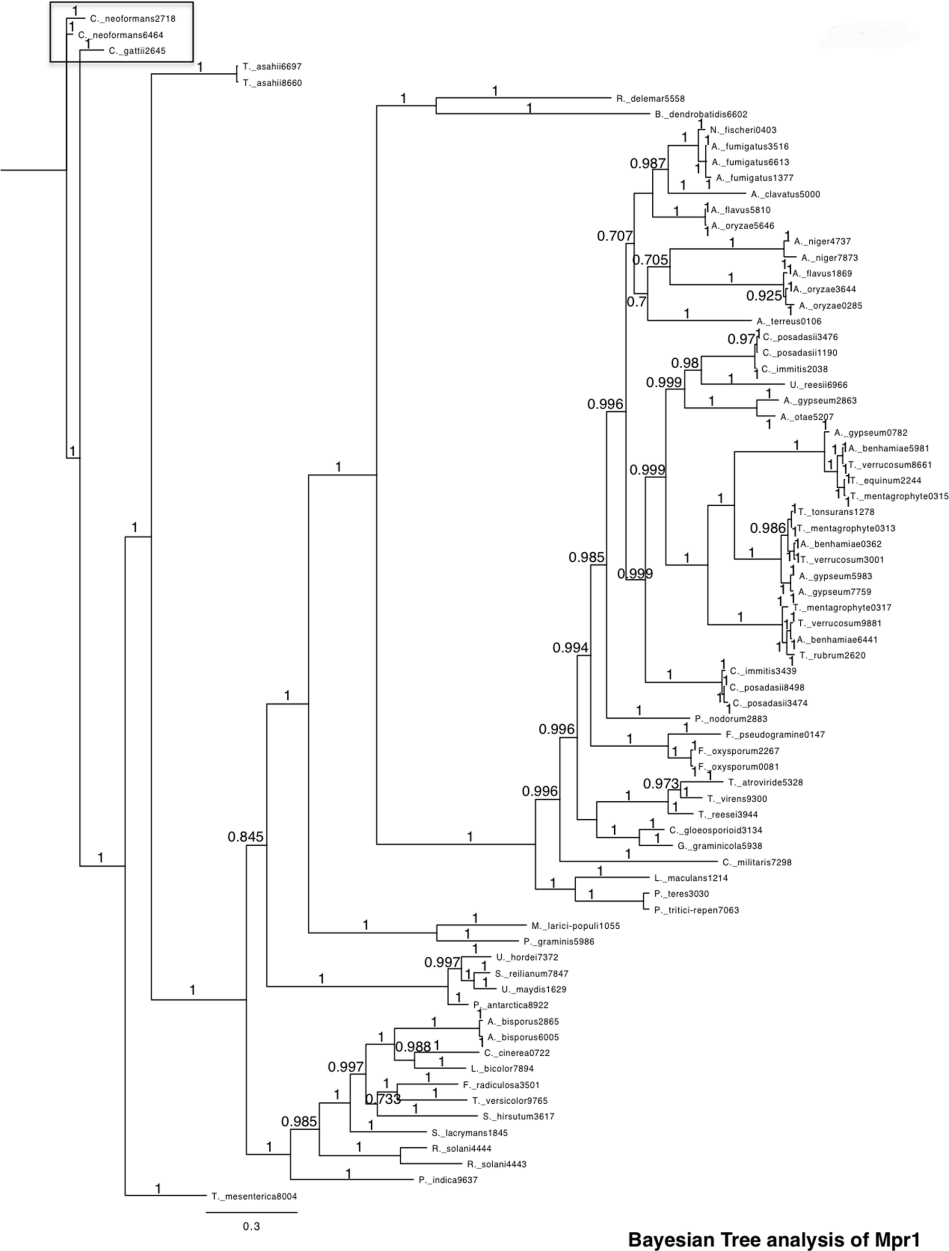
The MrBayes majority-rule consensus tree reveals a distinct phylogenetic relationship between Cryptococcal Mpr1 and other M36 family members. The MrBayes tree is based on ClustalW2 amino acid sequence alignments of CnMPR1 and other M36 family members and obtained from searching the HMMER web server (hmmer.janelia.org). The Bayesian tree reveals a distinct phylogenetic relationship between Mpr1 in Cryptococcus species and M36 family members in other fungi. An Mpr1 homolog was identified in the closely related basidiomycete *Tremella mesenterica*, however, Mpr1 clusters separately from the rest of the M36 family. Posterior probability values are shown as node labels. Well supported nodes indicated by a “1” with maximum likelihood bootstrap (BS) support >80.

We sought to further characterize the M36 class of metalloproteases and to specifically resolve the molecular mechanism of Mpr1 in *C*. *neoformans*. Based on the amino acid sequence alignment of Mpr1 across fungal species, a phylogenetic tree was constructed (Fig 1). The Bayesian tree analysis revealed a distinct phylogenetic relationship between Mpr1 in Cryptococcus species and the M36 fungalysin family members in other fungi (Fig 1). The Cryptococcus branch is farthest removed from all other fungi and most closely related to *Trichosporon asahii* and *Tremella mesenterica* (Fig 1).

### Amino acid substitution within the catalytic region of Mpr1 does not affect viability of Cryptococci.

**Fig 2:**
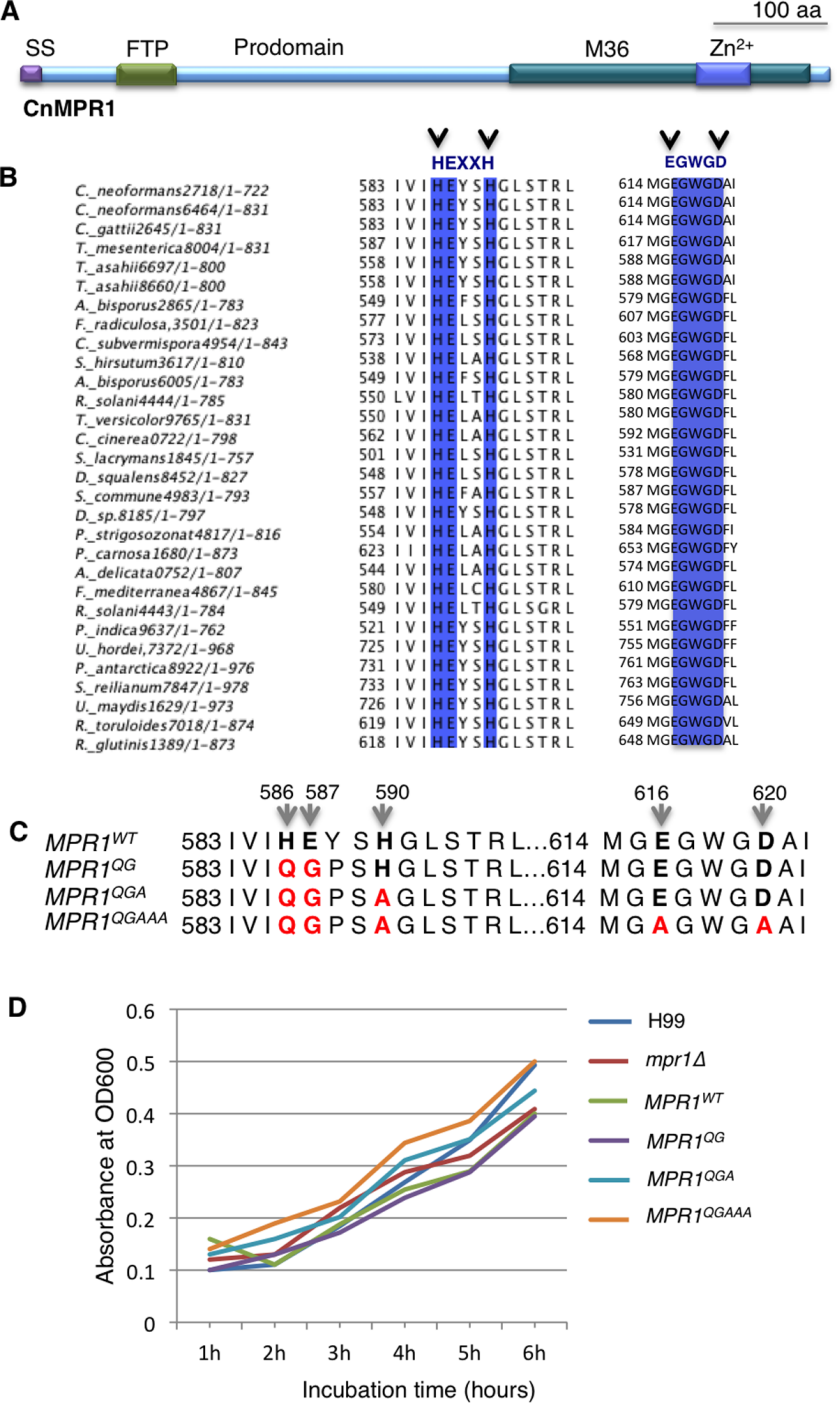
Substitution of conserved amino acids within regions of Mpr1 required for Zn^2+^ coordination and catalytic activity. **(A)** The scaled, schematic representation of the predicted structural features of Mpr1 indicates the signal sequence (SS), the prodomain containing a conserved FTP motif and the zinc-binding sites within the M36 catalytic domain. **(B)** The Mpr1 protein sequence alignment across fungal species revealed strong consensus among amino acids required for coordinating zinc and for catalytic activity (HExxH and ExxxD). (H, histidine; E, glutamic acid; D, aspartic acid). **(C)** Amino acid sequence depiction of wild type Mpr1 and three zinc-binding site mutants used in the study. *MPR1*^*wt*^ is the reconstituted strain (*mpr1Δ*::*MPR1*) with conserved H^586^E^587^xxH^590^ and E^616^xxxD^620^ regions. The *MPR1*^*QG*^ and *MPR1*^*QGA*^ mutants contain amino acid substitutions within the HExxH consensus region as follows: H^586^Q, and E^587^G or H^586^Q, E^587^G and H^590^A. The *MPR1*^*QGAAA*^ mutant contains substitutions in both HExxH and ExxxD consensus regions as follows: H^586^Q, E^587^G, H^590^A, E^616^A, D^620^A. (Q, glutamine; G, glycine; A, alanine). **(D)** The growth curves of *C*. *neoformans* strains expressing the *MPR1* mutants demonstrated no significant difference in the growth rates at 37°C suggesting that expression of the *MPR1* mutants did not negatively impact the viability of *C*. *neoformans*. The description of the *MPR1* mutants is detailed above. The H99 strain represents a wild type strain of *C*. *neoformans MPR1*^*WT*^. The *mpr1Δ* deletion strain (constructed in an H99 background) was used as the background strain for the expression of each *MPR1* mutant.

Mpr1 is a member of the M36 class of fungal metalloproteases and like other proteases within this group the predicted structure of Mpr1 from *C*. *neoformans* includes the typical hallmarks such as a signal sequence, a prodomain (containing an FTP motif) and a catalytic region at the C-terminus (Fig 2A). The presence of a signal sequence at the N-terminal region is consistent with the secretion of Mpr1 and is a defining feature of the M36 class.[18]

We demonstrated previously that Mpr1 was required for *C*. *neoformans* to cross the BBB and enter the central nervous system but whether the proteolytic activity of Mpr1 played a direct role in promoting the migration of fungal cells across the BBB was not known.[7, 30] Structure-function studies were performed in order to test the working hypothesis that mutations within the highly conserved regions of Mpr1 were central to Mpr1 activity. Here, site-directed mutagenesis was used to create single amino acid substitutions in the Mpr1 protein (S1 Sequences).

The catalytic region of Mpr1 contained two consensus sequences (HEXXH and EGWGD) that were highly conserved among fungal species (Fig 2B). Previous studies of glu-zincin, a group of metalloproteases that utilizes a zinc ion for catalytic activity, suggested that two histidines (H) of HExxH and the glutamic acid (E) of ExxxD coordinate with the metal ion during the catalytic process [33–36]. Following subcloning of the *MPR1* cDNA, histidine^586^ (H^586^) and glutamic acid^587^ (E^587^) within the consensus sequence (HEXXH), were replaced with glutamine (Q) and glycine (G), respectively (Fig 2C). By constructing the aforementioned mutant, *MPR1*^*QG*^, we aimed to determine the functional role of H^586^ and also E^587^, given that this particular glutamic acid was highly conserved.

In order to examine the functional contribution of the second histidine (H^590^) in the HEXXH consensus sequence, the *MPR1*^*QGA*^ mutant was constructed where H^590^ was replaced with alanine. The third and final mutant contained additional alanine substitutions in the glutamic acid (E^616^) and the aspartic acid (D^620^) of the ExxxD consensus sequence—*MPR1*^QGAAA.^ (Fig 2C). The *MPR1* mutants were transformed into the *mpr1Δ* deletion strain and Southern blot analysis and/or real time PCR confirmed one to two copies of *MPR1* mutant constructs were integrated (S1 Fig and S1 Data).

We found that despite the amino acid substitutions, the mutant strains grew robustly at 37°C with growth rates similar to the wild type H99 and the *MPR1* reconstituted strain (*mpr1Δ*::*MPR1*^*wt*^*)*(Fig 2D and S2 Data).

### Loss of proteolytic activity of Mpr1 prevents *C*. *neoformans* from crossing the BBB.

**Fig 3:**
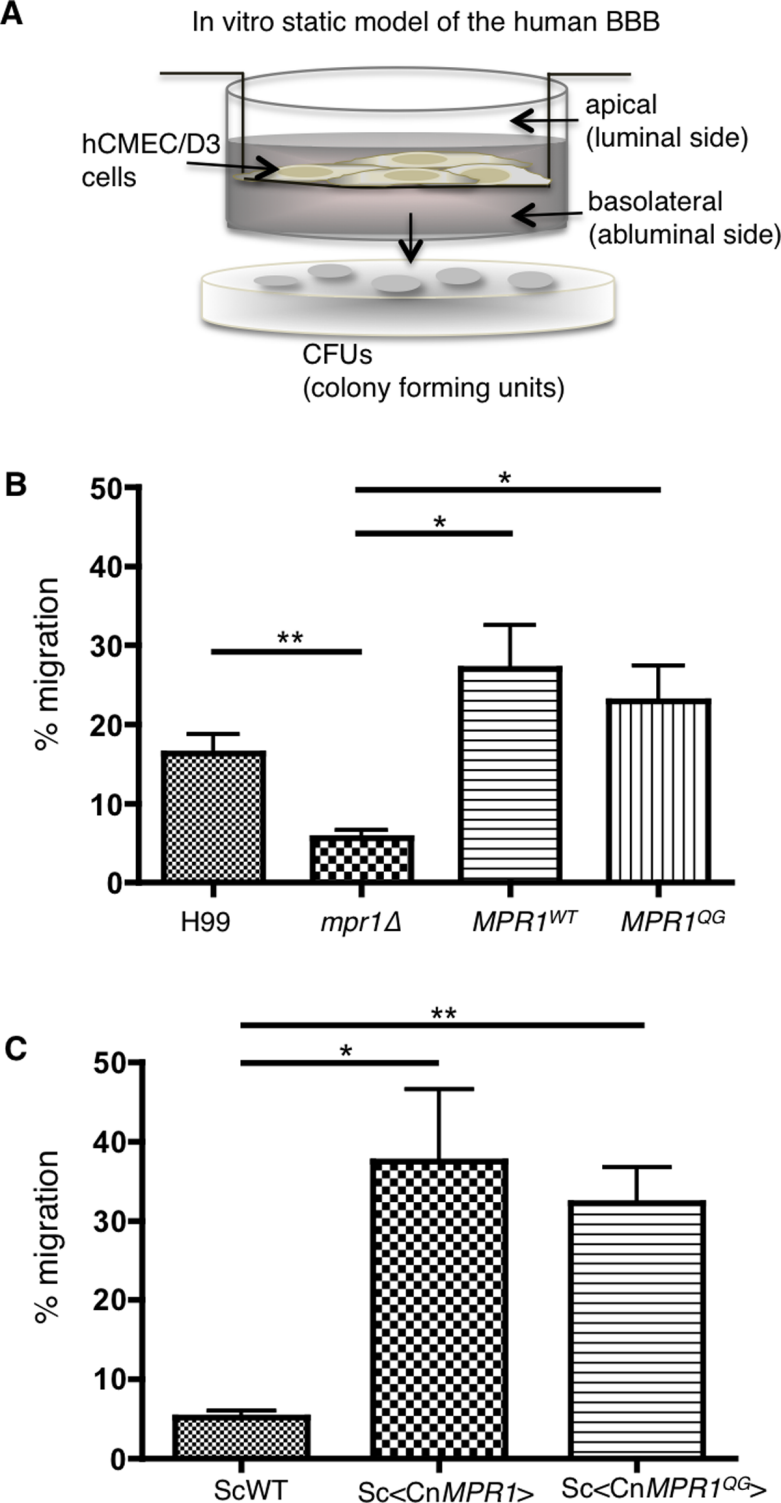
The amino acids, histidine^586^ and glutamic acid^587^ within the HExxH consensus region are not required for the transcellular migration of yeasts (*C*. *neoformans* and *S*. *cerevisiae)* across the BBB. **(A)** A schematic representation of the *in vitro*, static model of the human blood-brain barrier used in association- and transcytosis assays. An immortalized human brain capillary microvascular endothelial cell line (hCMEC/D3) is grown on collagen-coated transwells. A predetermined number of *C*. *neoformans* or *S*. *cerevisiae* yeast cells are added to the apical side (representing the blood-side of the BBB) of the transwell. Following migration across the human brain endothelial cells (a.k.a. the BBB), yeast cells are collected from the basolateral side (representing the brain-side of the BBB) of the transwells and plated for CFU determination. **(B)** A strain of *C*. *neoformans* expressing the *MPR1*^*QG*^ (H^586^Q, and E^587^G) mutant migrated across the BBB similar to the H99 parent strain and the Mpr1 reconstituted strain (*MPR1*^*WT*^) (*P > 0*.*05*, n = 5). **(C)** A wild type strain of *S*. *cerevisiae* expressing the wild type *MPR1* cDNA from *C*. *neoformans* (Sc<CnMPR1>), gained the ability to cross the BBB. Transcytosis assays of *S*. *cerevisiae* expressing the Cn*MPR1*^*QG*^ mutant revealed a similar transcellular migration as observed for Sc<CnMPR1> and consistent with results obtained for *C*. *neoformans*. (*P < 0*.*05*, n = 4). For the transcytosis assays reported in (B, C), yeast cells were collected from the basolateral side of the transwell following an 18h co-incubation of yeast with brain endothelial cells.

We hypothesized that substitution of amino acids coordinating zinc ion and mediating catalytic activity would affect Mpr1 function, thus we tested whether *C*. *neoformans* strains expressing *MPR1* mutants would display a defect in BBB crossing. Transcytosis assays using a static in vitro model of the human BBB were performed (Fig 3).[7, 29–32] Briefly, in this model, hCMEC/D3 cells (human brain capillary microvascular endothelial cells) were grown and allowed to differentiate on collagen-coated transwells submerged in media such that the upper chamber represented the luminal side (blood side of the BBB) and the lower chamber represented the abluminal side (brain side of the BBB) (Fig 3A). The integrity of the barrier was monitored by measuring transendothelial electrical resistance (TEER) or FITC-dextran permeability. Strains of *C*. *neoformans* were added to the luminal side of the transwell and subsequently the fungal cells that crossed the brain endothelium were collected at the abluminal side and plated on YPD agar for colony counting (Fig 3A).

The transcytosis assays revealed that the *C*. *neoformans* (Cn) *MPR1*^QG^ mutant strain crossed the barrier similar to the wild type strain (H99) and the reconstituted strain (*mpr1Δ*::*MPR1*^wt^) (Fig 3B). We also found that a strain of *S*. *cerevisiae* (Sc) expressing the CnMPR1^QG^ mutant cDNA retained its ability to cross the BBB. Generally, Sc cannot cross the BBB; however strains transformed with the plasmids carrying either CnMPR1^WT^ or CnMPR1^QG^ gained the ability to transmigrate the barrier (Fig 3C).[7, 30] Collectively the data suggest that the functional activity of Mpr1 was independent of the first histidine and glutamic acid in the HExxH conserved motif.

**Fig 4:**
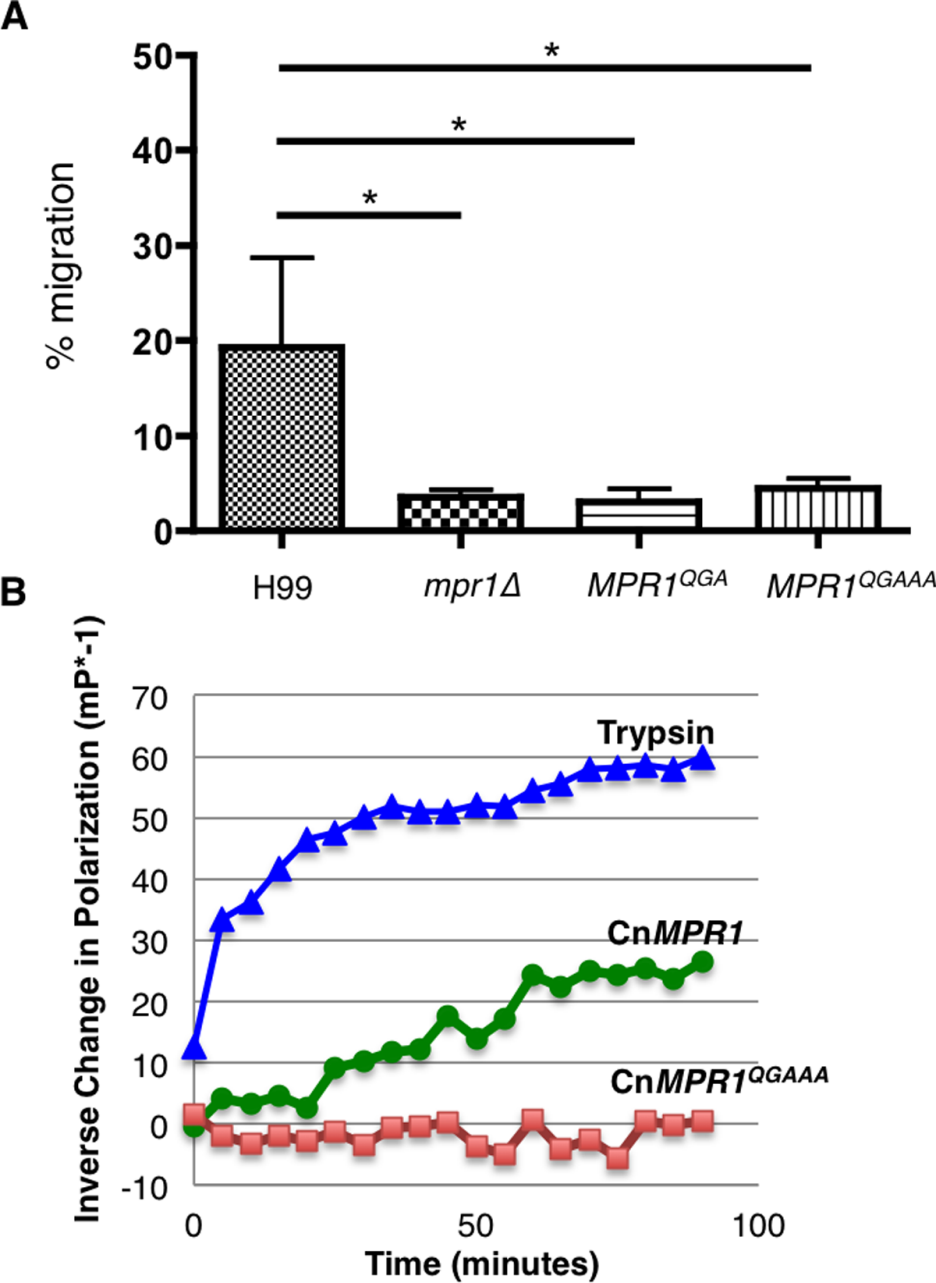
Amino acid substitution of all conserved residues within the HExxH and ExxxD motifs blocked proteolytic activity of Mpr1 and prevented *C*. *neoformans* from crossing the BBB. **(A)** The transcytosis assay showed that *C*. *neoformans* strains expressing MPR1 mutants with amino acid substitutions of all conserved residues in either HExxH (*MPR1*^*QGA*^) or HExxH and ExxxD (*MPR1*^*QGAAA*^) failed to cross the BBB in the *in vitro* model of BBB, in contrast to H99 (P < 0.05, n = 5). The *MPR1*^*QG*^ and *MPR1*^*QGA*^ mutants contain amino acid substitutions within the HExxH consensus region as follows: H^586^Q, and E^587^G or H^586^Q, E^587^G and H^590^A. The *MPR1*^*QGAAA*^ mutant contains substitutions in both HExxH and ExxxD consensus regions as follows: H^586^Q, E^587^G, H^590^A, E^616^A, D^620^A. (Q, glutamine; G, glycine; A, alanine). **(B)** The Cn*MPR1*^*QGAAA*^ mutant recombinant protein did not display proteolytic activity. The Cn*MPR1*^*QGAAA*^ mutant cDNA was expressed in a wild type strain of Sc (Sc<Cn*MPR1*^*QGAAA*^>) and recombinant mutant protein was isolated from the Sc<Cn*MPR1*^*QGAAA*^> strain. Cn*MPR1*^*QGAAA*^ recombinant protein displayed no proteolytic activity in contrast to wild type CnMpr1 recombinant protein (isolated from the Sc<Cn*MPR1*> strain). Proteolytic activity of Mpr1 was determined by FITC-casein fluorescent protease assay and trypsin was used as a positive control.

To examine whether the amino acid substitution of all the conserved residues within the HExxH and ExxxD motifs of Mpr1 would alter the ability of *C*. *neoformans* to cross the BBB, we performed transcytosis assays with a second set of Mpr1 mutants. We found that strains of *C*. *neoformans* expressing either the *MPR1*^QGA^ mutant or the *MPR1*^QGAAA^ mutant failed to cross the BBB similar to a strain of *C*. *neoformans* lacking *MPR1* (Fig 4A). To address whether BBB crossing was dependent on the proteolytic activity of Mpr1, we isolated recombinant Mpr1 protein from *S*. *cerevisiae* expressing CnMPR1^WT^ or CnMPR1^QGAAA^ via immobilized Ni^2+^ affinity chromatography (IMAC) and tested the proteolytic activity using a FITC-casein fluorescent protease assay.[7] As expected, we found that CnMPR1^WT^ recombinant protein maintained proteolytic function (Fig 4B, green line). In contrast, CnMPR1^QGAAA^ recombinant protein isolated from *S*. *cerevisiae*, showed no change in fluorescence polarization suggesting that the amino acid substitutions in both HExxH and ExxxD motifs abolished Mpr1 proteolytic activity (Fig 4B and S3 Data).

### Identification of the prodomain cleavage site for Mpr1

**Fig 5:**
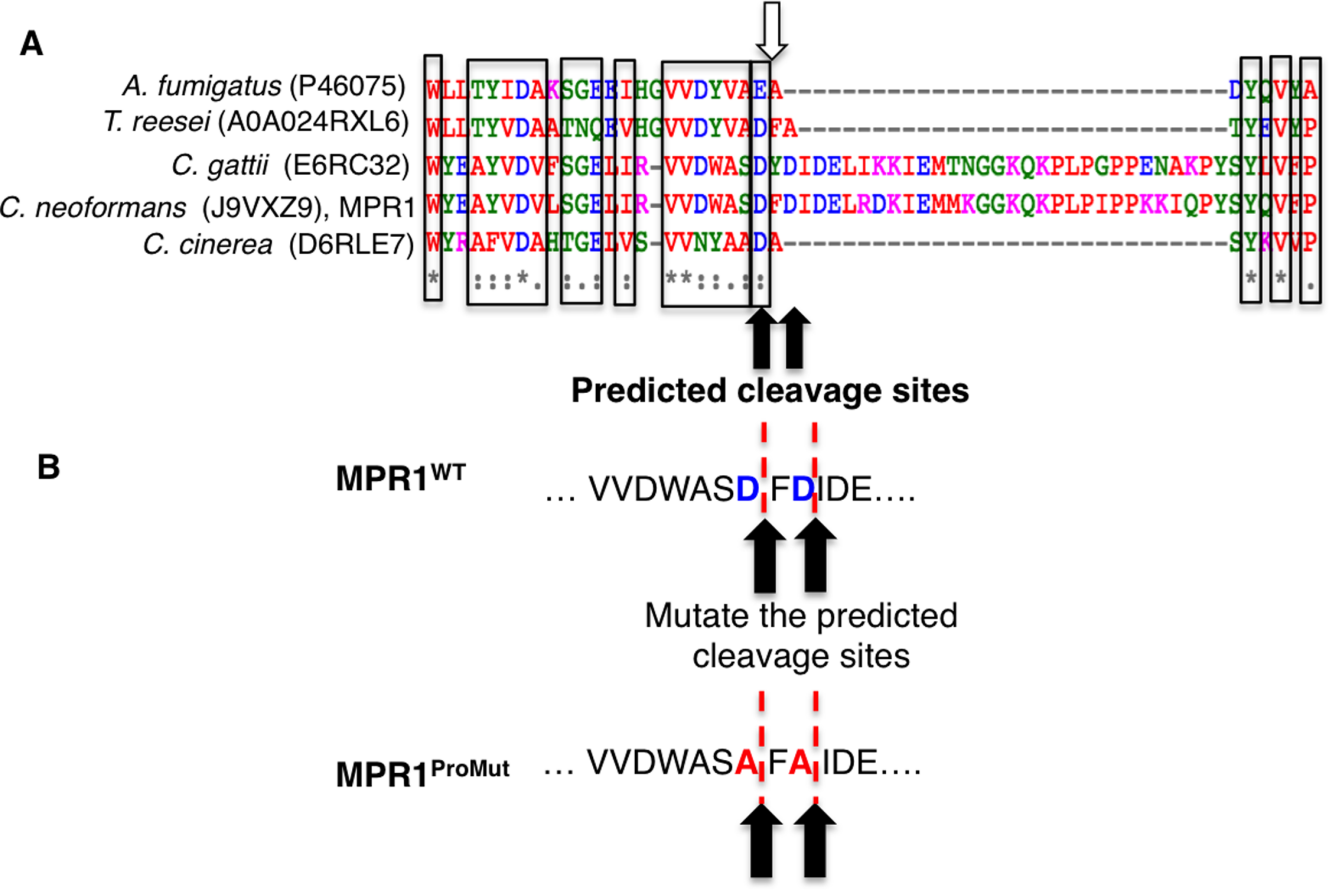
Multiple sequence alignment of known and predicted prodomains from members of the M36 family identified potential prodomain cleavage sites of Mpr1. **(A)** Peptide sequences of the M36 metalloproteases from *Trichoderma reesei*, *Cryptococcus gattii*, *Cryptococcus neoformans* (Mpr1), and *Coprinopsis cinerea* were aligned with the known cleavage site of *Aspergillus fumigatus* (white arrow) using MUSCLE sequence alignment (http://www.ebi.ac.uk/Tools/msa/muscle/). The results suggested two potential cleavage sites in the prodomain of Mpr1 at amino acid positions D^353^↓F^354^ and D^355^↓I^356^ (D: aspartic acid; F: phenylalanine; black arrows). **(B)** Two aspartic acids at the predicted cleavage sites (D^353^↓F^354^ and D^355^↓I^356^) of Mpr1 were mutated to alanine residues (A^353^↓F^354^ and A^355^↓I^356^). The red dotted lines indicate the proposed cleavage sites.

Many studies of proteases have revealed a conservation of amino acids surrounding cleavage sites of the prodomain [37–39]. An alignment comparing known cleavage sites of M36 metalloprotease from *A*. *fumigatus* [36] and other fungalysins including Mpr1 from *C*. *neoformans* was performed using MUSCLE multiple alignment (http://www.ebi.ac.uk/Tools/msa/muscle/) (Fig 5A). The result showed conserved amino acid residues at the predicted cleavage region among different fungalysins (Fig 5A). Based on the alignment and the known cleavage site of *A*. *fumigatus* M36 metalloprotease, we proposed two potential cleavage sites of cryptococcal Mpr1 at D^353^↓F^354^ and D^355^↓I^356^ (Fig 5B).

**Fig 6:**
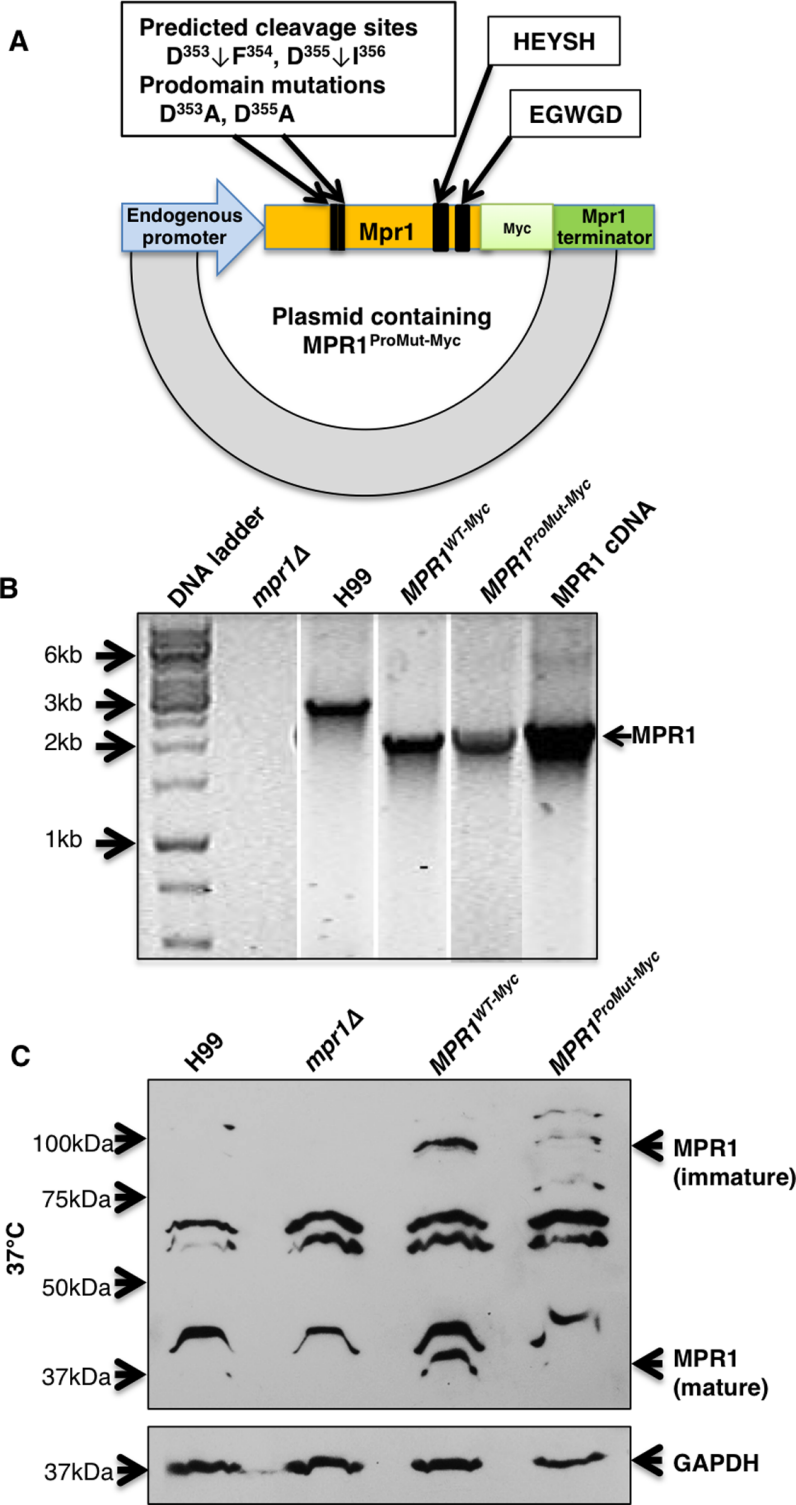
Transformation of the Mpr1 prodomain mutations into *mpr1Δ* strain shows the expression of Mpr1 at the protein level. **(A)** The plasmid vector containing Mpr1 with the point mutations (D^353^A and D^355^A) at the predicted cleavage sites was cloned into the *mpr1Δ* deletion strain. The endogenous promoter and terminator of Mpr1 was used to regulate the expression of Mpr1. A Myc-tag was added to the C-terminus of Mpr1 for protein detection. The wild type HExxH and ExxxD conserved motifs were kept intact in order to maintain autocatalytic activity. **(B)** Genomic DNA isolation and PCR confirmed the transformation of the Mpr1 prodomain mutations (MPR1^ProMut-Myc^) into the *mpr1Δ* strain. Bands at ~2.2 Kb, corresponding to the size of Mpr1 plus the Myc-tag, were detected in the reconstituted strain (*MPR1*^*WT-Myc*^) and the mutant strain (*MPR1*^*ProMut-Myc)*^) but not in *mpr1Δ* strain. The wild type H99 strain and a plasmid containing Mpr1 cDNA were used as controls for PCR. The genomic DNA of H99 encoding Mpr1 displayed a band size of ~2.7 kb as expected. **(C)** Western blot analysis using an anti-Myc-tag antibody revealed expression of Mpr1 in *MPR1*^*WT-Myc*^ and *MPR1*^*ProMut-Myc*^. Distinct bands at ~85 kDa and ~40 kDa, which correspond to the size of immature and mature forms of Mpr1 respectively, were observed in the reconstituted strain (*MPR1*^*WT-Myc*^). In contrast, the mature form of Mpr1 was not detected in the *MPR1*^*ProMu-tMyc*^ strain suggesting that the prodomain mutation prevented processing of a fully mature Mpr1. GAPDH was used as an loading control.

To test the role of these amino acids, the aspartic acid residues (D^353^ and D^355^) were substituted with alanine residues to create the prodomain mutant strain (MPR1^ProMut(Myc)^) for further study (Fig 5B). The plasmid containing *MPR1* cDNA with D^353^A and D^355^A substitutions and tagged with a MYC sequence at the C-terminus was transformed into a strain of *C*. *neoformans* lacking Mpr1 (*mpr1Δ*) (Fig 6A). The transformed colonies were confirmed by genomic DNA isolation and PCR. As expected a DNA band corresponding to *MPR1* appeared in the transformed strains but not in the *mpr1Δ* deletion strain (Fig 6B). Southern blot analysis revealed one copy of the *MPR1*^*Prodomain*^ mutant was integrated (S1 Fig and S1 Data).

### The mutagenesis of the Mpr1 prodomain cleavage site prevents maturation of Mpr1 protein

Western blot analysis of the *mpr1Δ* strain expressing MPR1^WT(Myc)^, revealed a polypeptide band at ~85kDa that corresponded to the predicted size of Mpr1 with its prodomain intact (i.e. mature form) (Fig 6C). Consistent with this, the 85kDa band was not detected in the *mpr1Δ* deletion strain or in the H99 strain transformed with empty plasmid. A smaller polypeptide band at ~40kDa was also detected in the MPR1^WT(Myc)^ strain but it was absent in the MPR1^ProMut(Myc)^ strain (Fig 6C). The 40kDa band was consistent with the predicted size of the Mpr1 protein lacking its prodomain (i.e. immature form) suggesting that the substitution of the two aspartic acid residues (D^353^ and D^355^) of the predicted prodomain cleavage site may have prevented the processing of the mature form of Mpr1. The data also suggest that aspartic acid residues D^353^ and D^355^ may represent a bona fide cleavage site of the prodomain.

### Mutagenesis of the Mpr1 prodomain cleavage site prevents fungal cells from crossing brain endothelial cells in the in vitro model of the BBB

**Fig 7:**
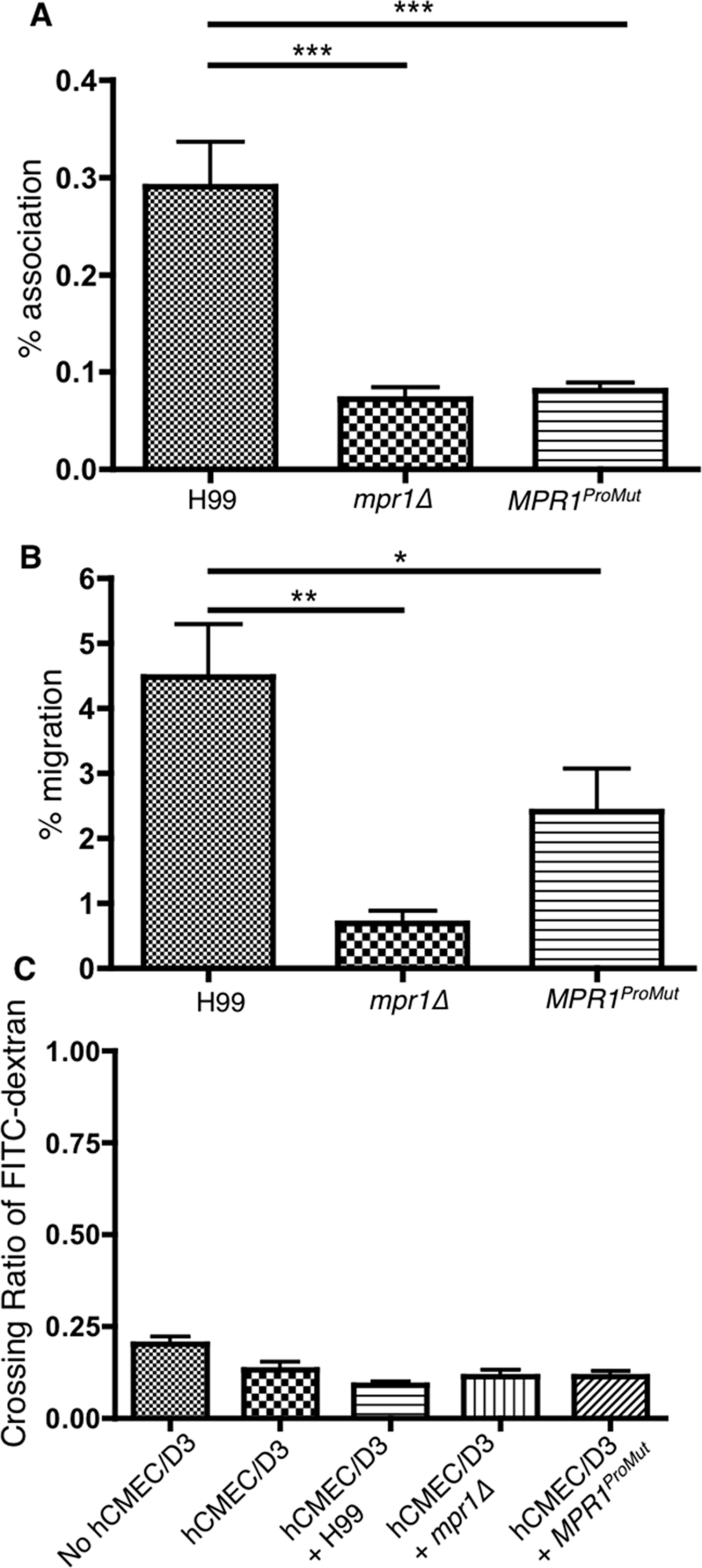
The expression of the *MPR1* mutations at sites D^353^ and D^355^ in *C*. *neoformans* reduces fungal association with brain endothelial cells and prevents fungal transcytosis across the BBB. **(A)** Following a 1h co-incubation of H99, *mpr1Δ*, or *MPR1*^*ProMut-Myc*^ strain with brain endothelial cells, association assays revealed a significant decrease in the adherence of the Cn*MPR1*^*ProMut-Myc*^ strain to the BBB (P< 0.05, n = 8). **(B)** Cryptococci expressing *MPR1*^*ProMut-Myc*^ displayed a significant decrease in BBB crossing in contrast to H99 and the *mpr1Δ* deletion strain (P<0.05, n = 8). **(C)** The integrity of the barrier in the vitro model of the human BBB remains intact during transcytosis assays. The FITC-dextran permeability assays revealed that the cellular junctions of the brain endothelial cells in the in vitro model of the BBB are not compromised during transcytosis assays indicated by the low FITC-dextran ratios (P> 0.05, n = 8).

In order to examine if the D^353^A, D^355^A substitutions of the prodomain cleavage site in Mpr1 altered the function of Mpr1, we tested the *MPR1*^ProMut(Myc)^ strain in the in vitro model of the human BBB. We first examined whether the Mpr1-prodomain mutant affected the direct association of fungal cells with the brain endothelial cells since we had previously determined that a complete lack of Mpr1 significantly reduced association of cryptococci and brain endothelial cells.[7] Following a 1 h incubation of the *MPR1*^ProMut(Myc)^ strain with brain endothelial cells in the BBB in vitro model, we found a significant reduction in the number of fungal cells associated with the BBB similar to the results observed for the *mpr1Δ* deletion strain. (Fig 7A) As expected, the H99 strain displayed a robust association with brain endothelial cells. (Fig 7A)

In a separate set of experiments, transcytosis assays performed at 6 h post-incubation of the *MPR1*^ProMut(Myc)^ strain with brain endothelial cells in the BBB in vitro model, revealed that significantly fewer *MPR1*^ProMut(Myc)^ cryptococci had crossed the BBB in contrast to the H99 strain but similar to the *mpr1Δ* deletion strain (Fig 7B). The integrity of the BBB was not significantly altered during the transcytosis assays (Fig 7C). Collectively, our observations were consistent with the notion that the mutation of the cleavage site within the prodomain prevents a functional Mpr1 protein likely due to the persistent attachment of the prodomain to the catalytic portion of Mpr1. In the absence of a functional Mpr1, *C*. *neoformans* was unable to cross the BBB.

## Discussion

Genome sequence analysis has revealed that not all fungi express fungalysins; for example *Saccharomyces cerevisiae* and *Neurospora crassa* have none while others like *Coprinopsis cinerea* have eight predicted fungalysins.[20, 40, 41] In the case of *Cryptococcus neoformans* only one fungalysin *MPR1*, is present in its genome.[7, 20] A phylogenetic tree based on the amino acid sequence alignment of Mpr1 across fungal species, revealed that Cryptococcus species share a distinct phylogenetic pattern in that its branch is farthest removed from all other fungi. This phylogenetic distinction may reflect the highly neurotropic nature of *C*. *neoformans* and the specific function of Mpr1 in breaching the blood-brain barrier.[7] The tree also revealed that the CnMpr1 is most closely related to the M36 fungalysin family member in *Trichosporon asahii* and *Tremella mesenterica*. Both share some antigens and are primarily opportunistic pathogens. *T*. *mesenterica* produces extracellular polysaccharides similar in composition and structure to those from *C*. *neoformans*. [42] *T*. *asahii* is urease positive and most often the cause of disseminated trichosporonosis in patients that are immunocompromised, similar to *C*. *neoformans*.[43]

The goal of this study was to perform a structure-function analysis of the Mpr1 protein with a particular focus on the predicted catalytic region of Mpr1 and the cleavage site of the prodomain. The predicted protein structure of Mpr1 consists of a signal peptide at its N-terminus adjacent to the FTP motif found in prodomains of M4 and M36 metalloproteases [44]. The catalytic region near the C-terminus consists of two highly conserved motifs HExxH and ExxxD, with predicted roles in zinc coordination. The presence of a signal peptide at the N-terminus is consistent with the secretion of Mpr1 and is a defining feature of the M36 class. We examined the role of the conserved amino acids in the HExxH and ExxxD motifs in relation to Mpr1 function emphasizing its role in the migration of *C*. *neoformans* through the brain endothelium.[7, 30] As we showed in the sequence alignment, the HExxH and ExxxD motifs have been preserved among fungal M36 metalloproteases implying a central role in protein function. Upon generating three mutant strains with various mutations in the HExxH and ExxxD motifs and testing their ability to cross the BBB, we found unexpectedly that the MPR1^QG^ strain demonstrated crossing ability similar to the wild type. The activity of MPR1^QG^ was confirmed in *S*. *cerevisiae* where the expression of CnMPR1^QG^ conferred crossing ability to *S*. *cerevisiae*. However, the MPR1^QGA^ and MPR1^QGAAA^ mutant strains were unable to cross the BBB, a likely consequence of a lack of catalytic activity.

The studies of the classes of metalloproteases containing HExxH and ExxxD motifs such as M4, M13, and M36 describe the formation of tetrahedral geometry surrounding zinc (Zn^2+^) [36, 45–47]. The two histidines of the HExxH motif and the glutamic acid of the ExxxD motif form three ligands and a water molecule serves as the fourth ligand. In thermolysin proteins (i.e. M4 metalloproteases) the water molecule interacts via a hydrogen bond with either the glutamic acid of the HExxH motif or a histidine residue located downstream of the ExxxD motif [47]. This alternative interaction suggests that even though the glutamic acid of HExxH is mutated, other residues could support function and in the case of Mpr1, this notion may explain the observed activity of the MPR1^QG^ mutant strain. Additionally, despite the lack of reports supporting glutamine (Q) as a known ligand in the catalytic site of metalloproteases [48], this amino acid has a polar side chain which could interact with a charged atom, so the first histidine substitution to glutamine in the MPR1^QG^ mutant strain might not abrogate Mpr1 function. However, the MPR1^QGA^ mutant strain, which had a similar mutation to MPR1^QG^ except that the second histidine of HExxH was substituted by an alanine residue, showed a significant defect in BBB crossing. This result is likely due to an impaired coordination between Zn^2+^ and alanine, which is considered a non-reactive amino acid.

For the ExxxD motif, unlike the glutamic acid that directly coordinates with Zn^2+^, the aspartic acid utilizes its carboxylate side chain to interact with the histidine of HExxH motif resulting in the formation of a Zn—H (from HExxH)—D (from ExxxD) triad [36, 49]. The crystallographic study of *A*. *fumigatus* metalloprotease (AfuMep), a fungalysin closely related to Mpr1, depicted a water channel made from hydrophilic amino acids including the aspartic acid of ExxxD (D^463^) coordinated to H^429^ (from H^429^ExxH motif) and this water path continues from the active site to the opposite surface of the protein [36]. It is worth mentioning that a polarized water molecule is responsible for nucleophilic attack resulting in cleavage of substrate peptides, thus the disruption of the water path by the mutation of the aspartic acid in ExxxD could lead to poor enzymatic activity consistent with the observed lack of proteolytic activity of the MPR1^QGAAA^ recombinant mutant and the inability of the MPR1^QGAAA^ mutant strain to cross the BBB.

The M36 class of fungalysins is found only in fungi and they are generally synthesized as pre-propeptides with fairly long prodomains (or propeptides).[50] Like other metalloproteases, fungalysins are likely processed into mature forms through the removal of the propeptide via an intracellular process that may involve enzymes sometimes found in the secretory pathway. The specific biological role of the FTP (fungalysin-thermolysin-propeptide) domain in the M36 class is still unclear but some studies suggest a role in protein folding, inhibition of enzyme activity, or involvement in autocatalytic activity [51–53].

An X-ray crystallographic analysis of the M36 fungalysin in *Aspergillus fumigatus* (AfuMep), suggested that cleavage of the prodomain began via an autoproteolytic break of the bond between the zinc-bound catalytic portion of AfuMep and the prodomain.[54] Final maturation of AfuMep would involve a stepwise degradation of the prodomain that subsequently would lead to the release of the mature AfuMep. [54] Our study identified the prodomain cleavage sites of Mpr1 and demonstrated that when mutated, the prodomain appears to remain attached to the catalytic C-terminus of Mpr1 rendering a nonfunctional Mpr1 protein. Some fungal metalloproteases can be specifically inhibited by their propeptides.[55] In the case of Mpr1, the specific role of the prodomain remains an open question. An attempt to thread the amino acid sequence of Mpr1 onto the structure AfuMep revealed less than optimal models and thus precluded its inclusion in this study, but it would suggest that some significant differences between Mpr1 and AfuMep likely exist.

Collectively, the data presented in this study contribute to a deeper understanding of the regulation of Mpr1—an important virulence factor in the pathogenesis of *C*. *neoformans*. By resolving the molecular determinants of Mpr1 regulation and activity we can now begin to identify specific inhibitors that could potentially be used to block fungal penetration of the CNS and thus prevent cryptococcal meningoencephalits-related deaths.

## Supporting information

S1 Data Real time PCR analysis of *MPR1* mutant gene copy number. (XLSX)

S2 Data Cell growth assays. (DOCX)

S3 Data Proteolytic activities of Mpr1 mutant strains. (XLSX)

S1 Sequences Amino acid sequences of Mpr1 mutants. (DOCX)

S1 Fig Determination of genomic *MPR1* mutant copy number in proteolytic and prodomain mutant strains. **(A)** Southern blot analysis detected a single *MPR1* copy number in MPR1^wt^, MPR1^QG^ and MPR1^Prodomain^ transformed strains, suggesting mutant constructs were integrated as single copy. **(B)** Despite the negative Southern blot result for the remaining strains, the presence of *MPR1* gene in MPR1^QGA^ and MPR1^QGAAA^ strains is confirmed by PCR. Q solution (Qiagen) is a PCR enhancing additive. **(C)** Real time PCR confirmed 1 to 2 copies of genomic *MPR1* mutant in MPR1^QGA^ and MPR1^QGAAA^ strains. Real time PCR was performed with 2 different sets of primers.
(TIFF)
