# Supplementary material for: The structure-function analysis of the Mpr1 metalloprotease determinants of activity during migration of fungal cells across the blood-brain barrier
Source: PLoS One. 2018 Aug 30;13(8):e0203020. doi: 10.1371/journal.pone.0203020 (PMC6117016; doi:10.1371/journal.pone.0203020)
Supplement: S2 Data — (DOCX) [file pone.0203020.s002.docx]

**Supplementary**

1. Raw Data for Figure 3B


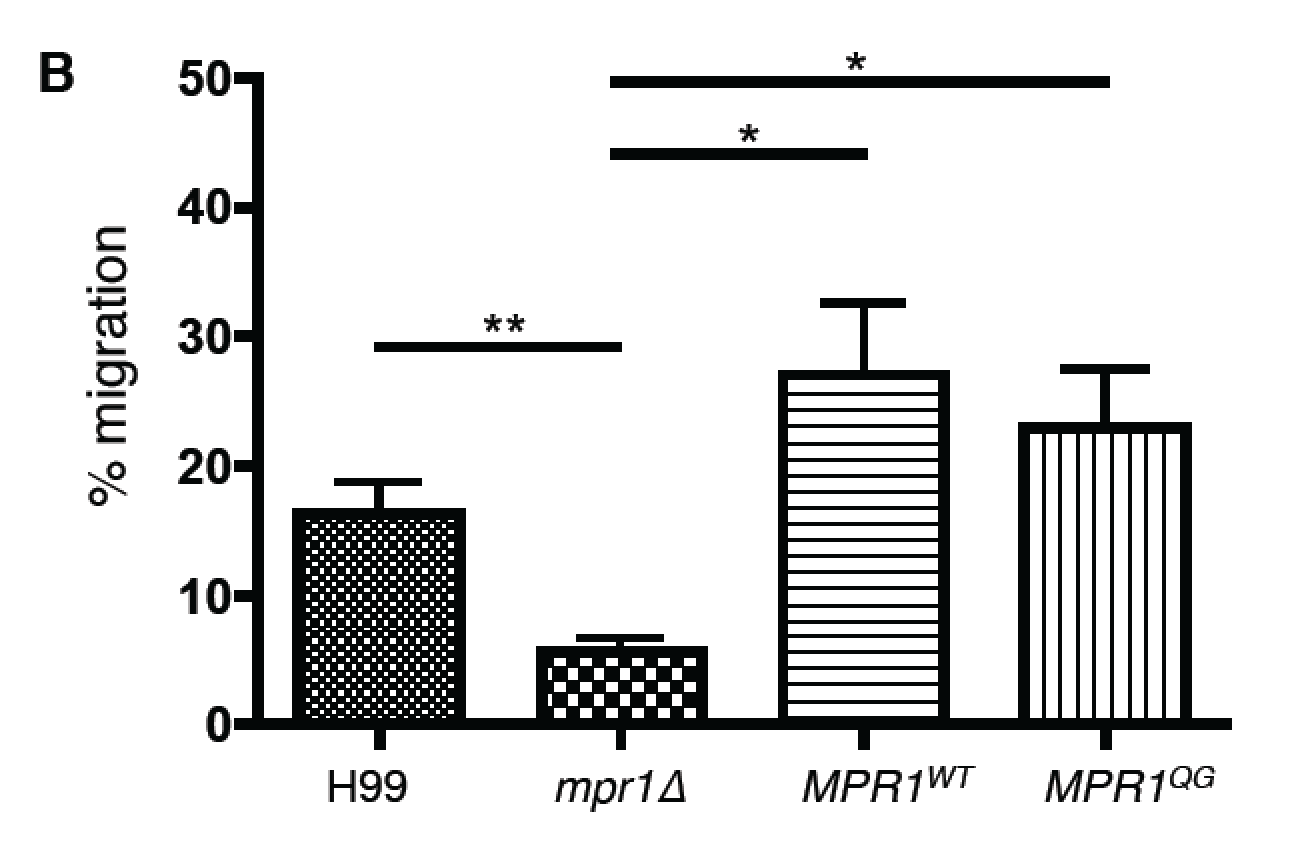


| **Replicate**  **Number** | **Strain** | | | | | | | |
| --- | --- | --- | --- | --- | --- | --- | --- | --- |
|  | **H99** | | ***mpr1Δ*** | | ***MPR1^WT^*** | | ***MPR1^QG^*** | |
|  | **CFUs** | **% migration** | **CFUs** | **%**  **migration** | **CFUs** | **%**  **migration** | **CFUs** | **%**  **migration** |
| 1 | 2.27 x 10^5^ | 22.70 | 7.10 x 10^4^ | 7.10 | 2.63 x 10^5^ | 26.30 | 2.58 x 10^5^ | 25.80 |
| 2 | 1.70 x 10^5^ | 17.00 | 3.20 x 10^4^ | 3.20 | 3.15 x 10^5^ | 31.50 | 3.88 x 10^5^ | 38.80 |
| 3 | 1.53 x 10^5^ | 15.30 | 8.50 x 10^4^ | 8.50 | 3.82 x 10^5^ | 38.20 | 1.87 x 10^5^ | 18.70 |
| 4 | 1.88 x 10^5^ | 18.80 | 6.00 x 10^4^ | 6.00 | 1.21 x 10^5^ | 12.10 | 1.90 x 10^5^ | 19.00 |
| 5 | 7.90 x 10^4^ | 7.90 | 3.30 x 10^4^ | 3.30 | 5.80 x 10^4^ | 5.80 | 1.25 x 10^5^ | 12.50 |

Note: % migration = $\frac{CFUs}{{10}^{6}} \times100$

10^6^ is the number of yeast cells added into transwells at the starting time point.

1. Raw Data for Figure 3C


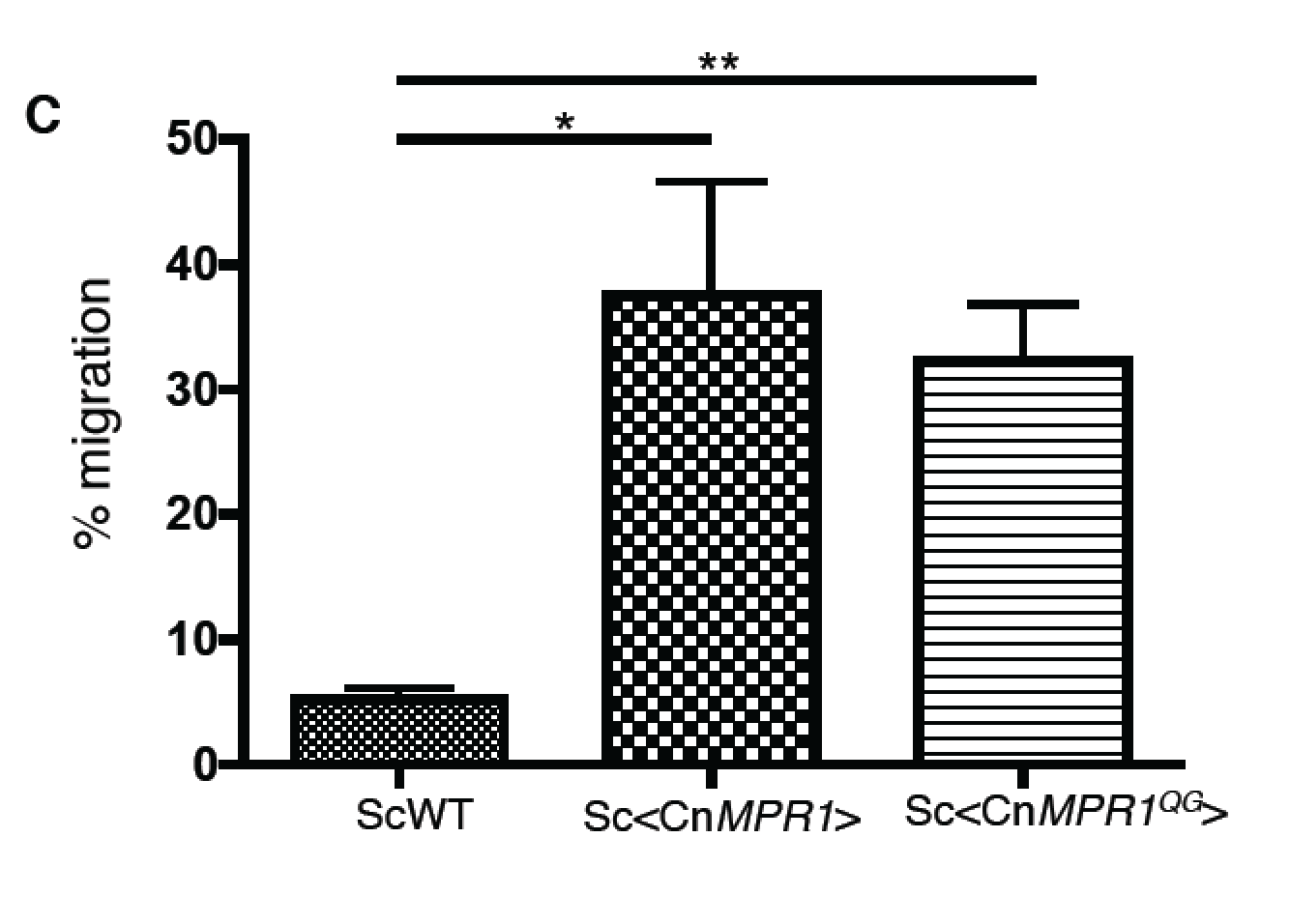


| **Replicate**  **Number** | **Strain** | | | | | |
| --- | --- | --- | --- | --- | --- | --- |
|  | **ScWT** | | **Sc<CnMPR1>** | | **Sc< Cn*MPR1^QG^* >** | |
|  | **CFUs** | **% migration** | **CFUs** | **%**  **migration** | **CFUs** | **%**  **migration** |
| 1 | 7.60 x 10^4^ | 7.60 | 1.80 x 10^5^ | 18.00 | 4.30 x 10^5^ | 43.00 |
| 2 | 5.04 x 10^4^ | 5.04 | 6.20 x 10^5^ | 62.00 | 2.00 x 10^5^ | 20.00 |
| 3 | 3.56 x 10^4^ | 3.56 | 3.40 x 10^5^ | 34.00 | 3.30 x 10^5^ | 33.00 |
| 4 | 4.48 x 10^4^ | 4.48 | 3.60 x 10^5^ | 36.00 | 2.20 x 10^5^ | 22.00 |

Note: % migration = $\frac{CFUs}{{10}^{6}} \times100$

10^6^ is the number of yeast cells added into transwells at the starting time point.

1. Raw Data for Figure 4A


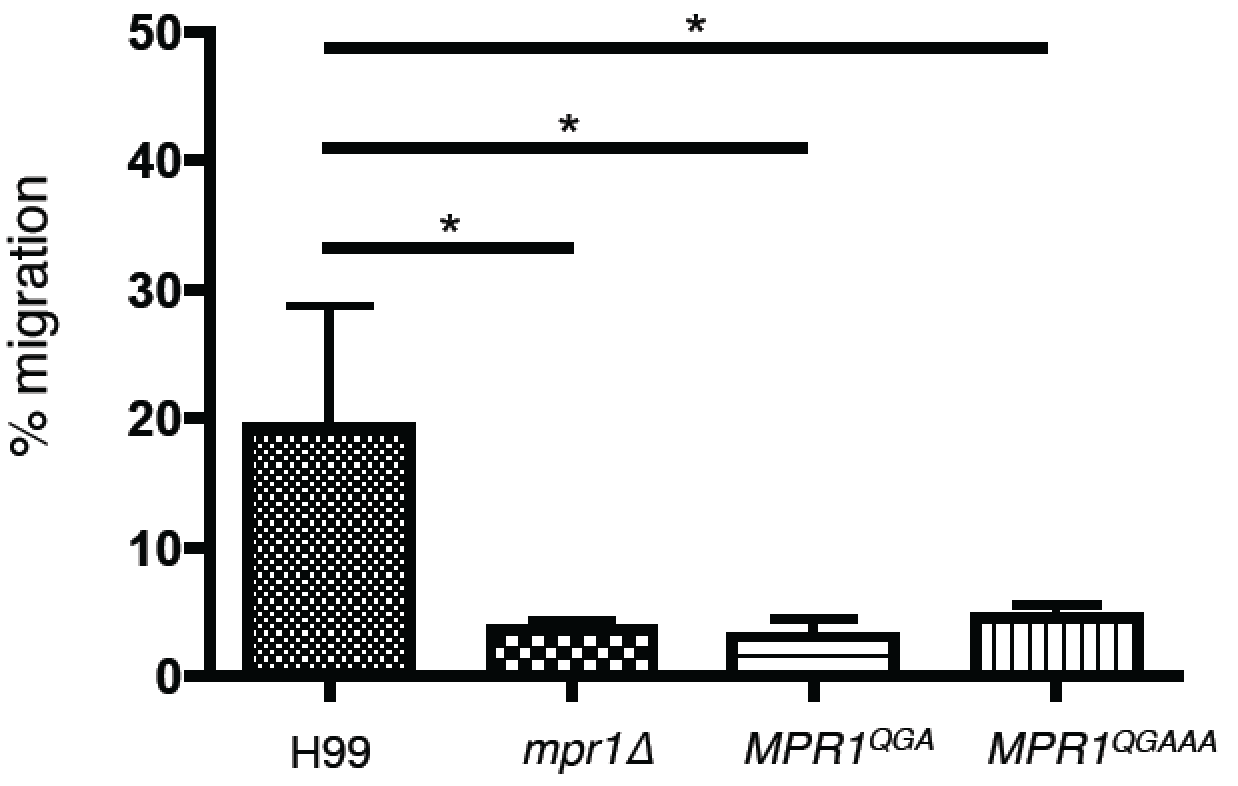


| **Replicate**  **Number** | **Strain** | | | | | | | |
| --- | --- | --- | --- | --- | --- | --- | --- | --- |
|  | **H99** | | ***mpr1Δ*** | | ***MPR1^QGA^*** | | ***MPR1^QGAAA^*** | |
|  | **CFUs** | **% migration** | **CFUs** | **%**  **migration** | **CFUs** | **%**  **migration** | **CFUs** | **%**  **migration** |
| 1 | 1.40 x 10^5^ | 14.00 | 2.70 x 10^4^ | 2.70 | 6.40 x 10^3^ | 0.64 | 4.50 x 10^4^ | 4.50 |
| 2 | 8.40 x 10^4^ | 8.40 | 4.80 x 10^4^ | 4.80 | 7.40 x 10^4^ | 7.40 | 2.90 x 10^4^ | 2.90 |
| 3 | 7.50 x 10^4^ | 7.50 | 5.80 x 10^4^ | 5.80 | 5.00 x 10^3^ | 0.50 | 4.10 x 10^4^ | 4.10 |
| 4 | 1.52 x 10^5^ | 15.20 | 3.20 x 10^4^ | 3.20 | 2.50 x 10^4^ | 2.50 | 3.20 x 10^4^ | 3.20 |
| 5 | 3.21 x 10^5^ | 32.10 | 1.50 x 10^4^ | 1.50 | 4.60 x 10^4^ | 4.60 | 4.80 x 10^4^ | 4.80 |

Note: % migration = $\frac{CFUs}{{10}^{6}} \times100$

10^6^ is the number of yeast cells added into transwells at the starting time point.

1. Raw Data for Figure 7A


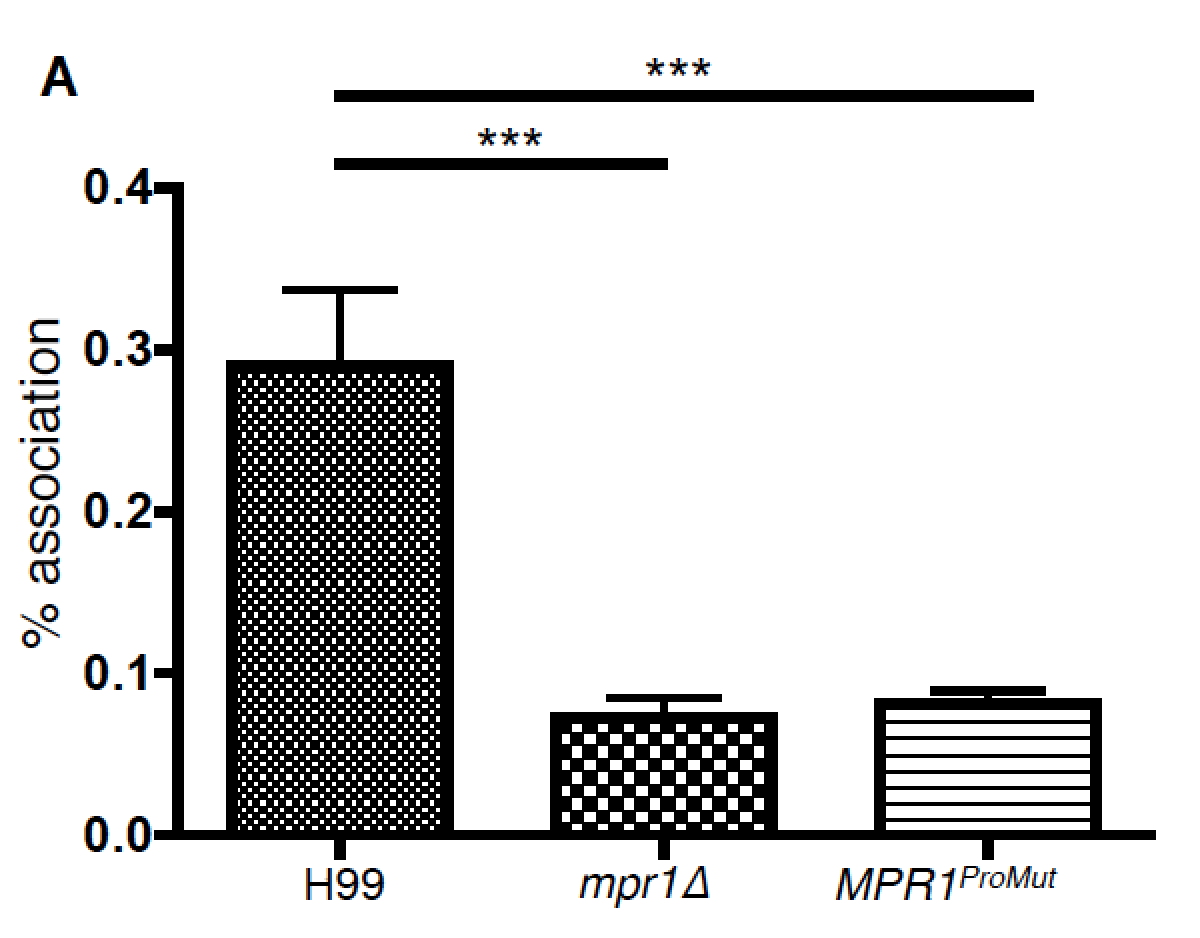


| **Replicate**  **Number** | **Strain** | | | | | |
| --- | --- | --- | --- | --- | --- | --- |
|  | **H99** | | ***mpr1Δ*** | | ***MPR1^ProMut^*** | |
|  | **CFUs** | **% association** | **CFUs** | **% association** | **CFUs** | **% association** |
| 1 | 3.00 x 10^3^ | 0.300 | 6.10 x 10^2^ | 0.061 | 7.50 x 10^2^ | 0.075 |
| 2 | 4.20 x 10^3^ | 0.420 | 1.33 x 10^3^ | 0.133 | 6.40 x 10^2^ | 0.064 |
| 3 | 2.90 x 10^3^ | 0.290 | 9.60 x 10^2^ | 0.096 | 9.50 x 10^2^ | 0.095 |
| 4 | 2.20 x 10^3^ | 0.220 | 8.00 x 10^2^ | 0.080 | 1.14 x 10^3^ | 0.114 |
| 5 | 1.86 x 10^3^ | 0.186 | 8.50 x 10^2^ | 0.085 | 6.50 x 10^2^ | 0.065 |
| 6 | 1.22 x 10^3^ | 0.122 | 3.30 x 10^2^ | 0.033 | 1.10 x 10^3^ | 0.110 |
| 7 | 5.30 x 10^3^ | 0.530 | 5.30 x 10^2^ | 0.053 | 7.20 x 10^2^ | 0.072 |
| 8 | 2.58 x 10^3^ | 0.258 | 3.70 x 10^2^ | 0.037 | 5.30 x 10^2^ | 0.053 |

Note: % association = $\frac{CFUs}{{10}^{6}} \times100$

10^6^ is the number of yeast cells added into transwells at the starting time point.

1. Raw Data for Figure 7B


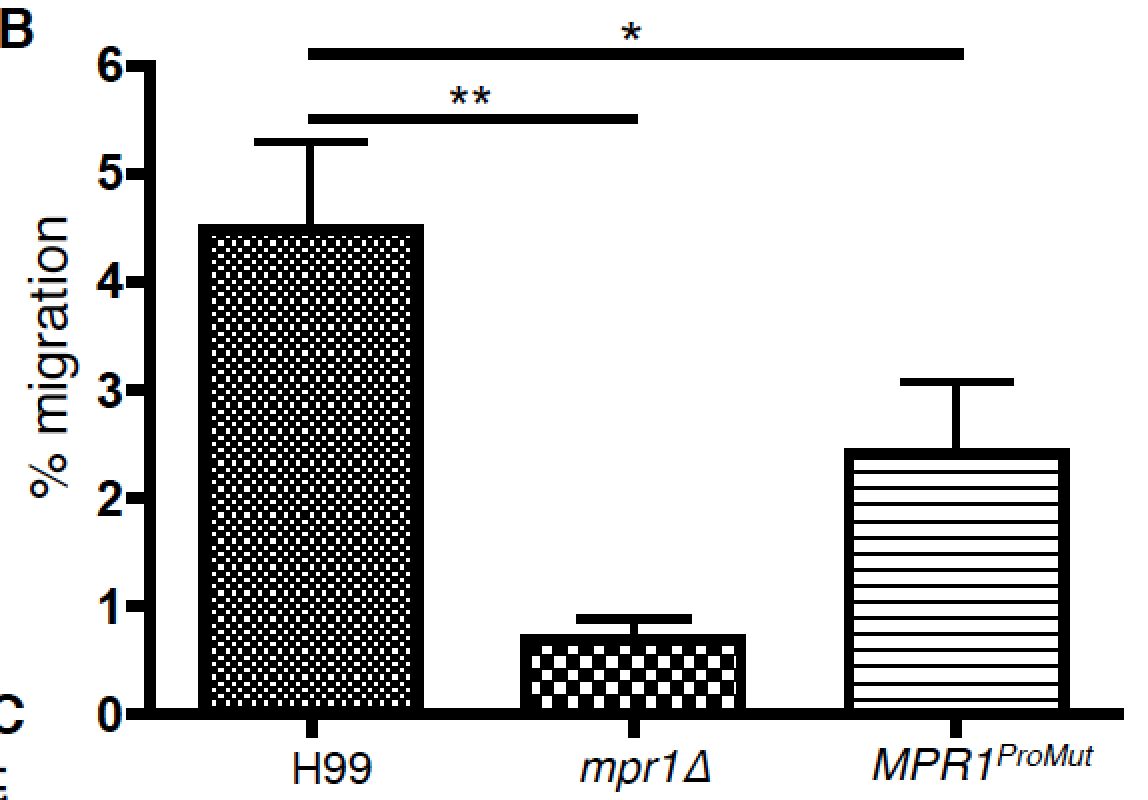


| **Replicate**  **Number** | **Strain** | | | | | |
| --- | --- | --- | --- | --- | --- | --- |
|  | **H99** | | ***mpr1Δ*** | | ***MPR1^ProMut^*** | |
|  | **CFUs** | **% migration** | **CFUs** | **% migration** | **CFUs** | **% migration** |
| 1 | 4.70 x 10^4^ | 4.70 | 3.30 x 10^3^ | 0.33 | 1.00 x 10^3^ | 0.10 |
| 2 | 2.20 x 10^4^ | 2.20 | 8.90 x 10^3^ | 0.89 | 2.00 x 10^3^ | 0.20 |
| 3 | 1.90 x 10^4^ | 1.90 | 9.90 x 10^3^ | 0.99 | 4.00 x 10^3^ | 0.40 |
| 4 | 3.90 x 10^4^ | 3.90 | 1.14 x 10^4^ | 1.14 | 3.20 x 10^4^ | 3.20 |
| 5 | 4.50 x 10^4^ | 4.50 | 6.80 x 10^3^ | 0.68 | 3.00 x 10^4^ | 3.00 |
| 6 | 7.90 x 10^4^ | 7.90 | 2.00 x 10^3^ | 0.20 | 1.80 x 10^4^ | 1.80 |
| 7 | 2.50 x 10^4^ | 2.50 | 8.50 x 10^3^ | 0.85 | 2.10 x 10^4^ | 2.10 |
| 8 | 8.80 x 10^4^ | 8.80 | 3.90 x 10^3^ | 0.39 | 7.10 x 10^4^ | 7.10 |

Note: % migration = $\frac{CFUs}{{10}^{6}} \times100$

10^6^ is the number of yeast cells added into transwells at the starting time point.

1. Raw Data for Figure 7C


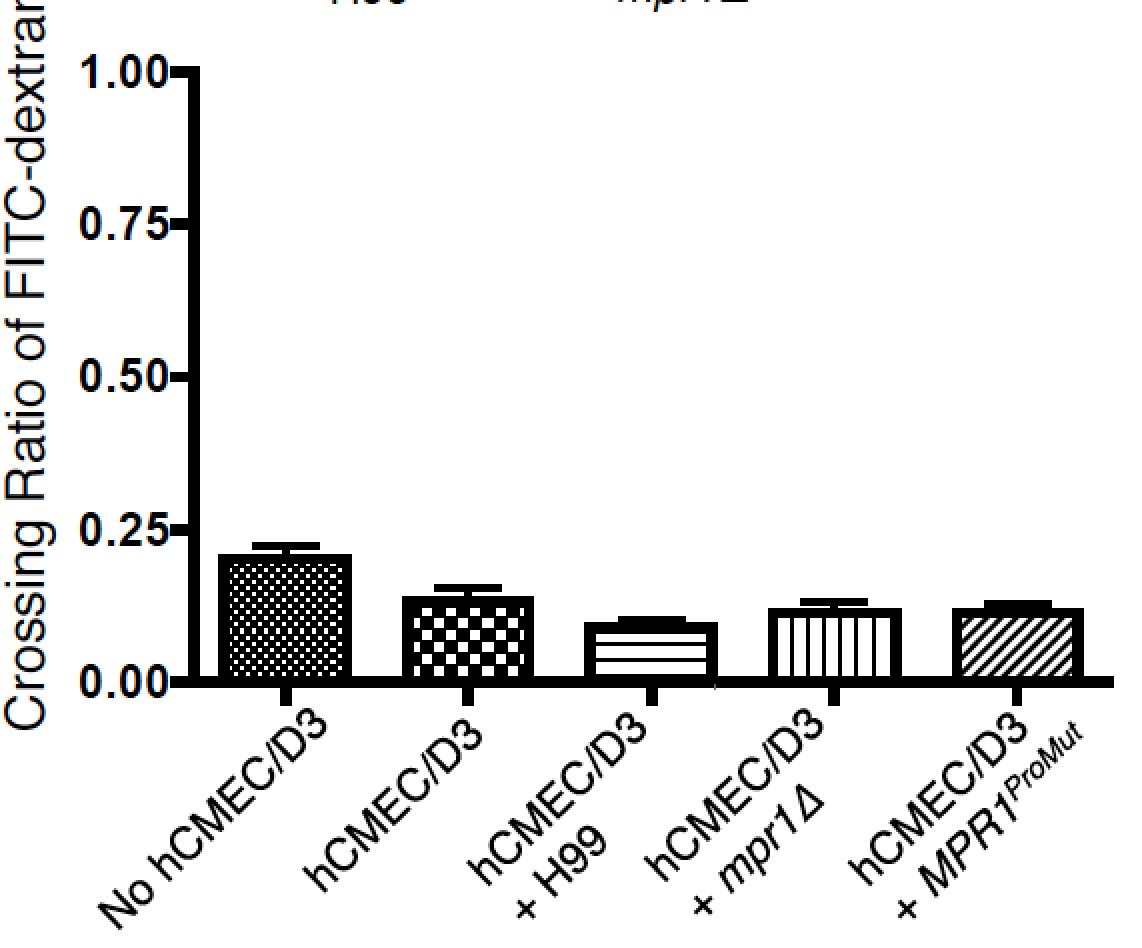


| **Replicate**  **Number** | **Crossing Ratio of FITC-dextran** | | | | |
| --- | --- | --- | --- | --- | --- |
|  | **No hCMEC/D3** | **hCMEC/D3** | **hCMEC/D3 +**  **H99** | **hCMEC/D3 + *mpr1Δ*** | **hCMEC/D3 + *MPR1^ProMut^*** |
| 1 | 0.2215 | 0.1529 | 0.0812 | 0.1579 | 0.1181 |
| 2 | 0.1825 | 0.1525 | 0.0809 | 0.0787 | 0.1798 |
| 3 | 0.2018 | 0.0889 | 0.0556 | 0.0849 | 0.1148 |
| 4 | 0.2679 | 0.1234 | 0.0993 | 0.0811 | 0.1034 |
| 5 | 0.1782 | 0.1632 | 0.1013 | 0.1033 | 0.0605 |
| 6 | 0.1642 | 0.1167 | 0.1236 | 0.1776 | 0.1037 |
| 7 | 0.2532 | 0.1054 | 0.0876 | 0.0986 | 0.1365 |
| 8 | 0.1457 | 0.1485 | 0.0930 | 0.1292 | 0.0903 |
