# Supplementary material for: The structure-function analysis of the Mpr1 metalloprotease determinants of activity during migration of fungal cells across the blood-brain barrier
Source: PLoS One. 2018 Aug 30;13(8):e0203020. doi: 10.1371/journal.pone.0203020 (PMC6117016; doi:10.1371/journal.pone.0203020)
Supplement: S1 Sequences — (DOCX) [file pone.0203020.s004.docx]

***MPR1-WT***

1 mrssaliall pflatltaar phhredkhsa srtrkslsfg pahshasfev lddavhvfep

61 rglidepidv krvaqtflgs qlgaqegegf yiredsytda rtgvthifar qllnglevsd

121 gdinlnidrd grimswgnsf hpgsvpslsd ihssssgete kvcttlhqhl dehkahlael

181 kgetgiwglv ksaaqvvlgs slplgevdhh eikethksmr hienhlramc dqpavstqsm

241 lspvealvsl lprlspiddl edispfdlts tphhtlkpkp afaepptevi sgaalskagv

301 vsdvsarlmy tqvsegaprl vwkyevemkd swyeayvdvl sgelirvvdw asdfdidelr

361 dkiemmkggk qkplpippkk iqpysyqvfp wgindpvsgn lsvvtepwdt vasplgwhsf

421 ptsanpwdvt ipgettnhny tvfnttagnn vyahenwegr nnfllnyrpt ndshifvyey

481 gepeglapke yvdmvvtqlf ytanmyhdll yrlgfdelsg nfqaynfrlg gkggdpvvcn

541 aqdgsgynna nfltppdgqa prmrmyiwdt atpyrdgdle agivi**HEYSH** glstrltggp

601 ansgclgyge aggmg**EGWGD** aiatlirqve ehknfengtd vfsmgawaan snrgirnyky

661 stnftinpst yktldkpgyw gvhaigevwa eflfvlsqrl vekygfgptl fpptdtskhn

721 dyytrtsees vdaagrplpl vpkygnalai qlivdamklq pcrpsffdar naiiqadqil

781 tggenacliw qafaerglge daavvgqtpw gggvrsdgfk vpkkvceskk a

***MPR1-QG***

1 mrssaliall pflatltaar phhredkhsa srtrkslsfg pahshasfev lddavhvfep

61 rglidepidv krvaqtflgs qlgaqegegf yiredsytda rtgvthifar qllnglevsd

121 gdinlnidrd grimswgnsf hpgsvpslsd ihssssgete kvcttlhqhl dehkahlael

181 kgetgiwglv ksaaqvvlgs slplgevdhh eikethksmr hienhlramc dqpavstqsm

241 lspvealvsl lprlspiddl edispfdlts tphhtlkpkp afaepptevi sgaalskagv

301 vsdvsarlmy tqvsegaprl vwkyevemkd swyeayvdvl sgelirvvdw asdfdidelr

361 dkiemmkggk qkplpippkk iqpysyqvfp wgindpvsgn lsvvtepwdt vasplgwhsf

421 ptsanpwdvt ipgettnhny tvfnttagnn vyahenwegr nnfllnyrpt ndshifvyey

481 gepeglapke yvdmvvtqlf ytanmyhdll yrlgfdelsg nfqaynfrlg gkggdpvvcn

541 aqdgsgynna nfltppdgqa prmrmyiwdt atpyrdgdle agivi**QGYSH** glstrltggp

601 ansgclgyge aggmg**EGWGD** aiatlirqve ehknfengtd vfsmgawaan snrgirnyky

661 stnftinpst yktldkpgyw gvhaigevwa eflfvlsqrl vekygfgptl fpptdtskhn

721 dyytrtsees vdaagrplpl vpkygnalai qlivdamklq pcrpsffdar naiiqadqil

781 tggenacliw qafaerglge daavvgqtpw gggvrsdgfk vpkkvceskk a

***MPR1-QGA***

1 mrssaliall pflatltaar phhredkhsa srtrkslsfg pahshasfev lddavhvfep

61 rglidepidv krvaqtflgs qlgaqegegf yiredsytda rtgvthifar qllnglevsd

121 gdinlnidrd grimswgnsf hpgsvpslsd ihssssgete kvcttlhqhl dehkahlael

181 kgetgiwglv ksaaqvvlgs slplgevdhh eikethksmr hienhlramc dqpavstqsm

241 lspvealvsl lprlspiddl edispfdlts tphhtlkpkp afaepptevi sgaalskagv

301 vsdvsarlmy tqvsegaprl vwkyevemkd swyeayvdvl sgelirvvdw asdfdidelr

361 dkiemmkggk qkplpippkk iqpysyqvfp wgindpvsgn lsvvtepwdt vasplgwhsf

421 ptsanpwdvt ipgettnhny tvfnttagnn vyahenwegr nnfllnyrpt ndshifvyey

481 gepeglapke yvdmvvtqlf ytanmyhdll yrlgfdelsg nfqaynfrlg gkggdpvvcn

541 aqdgsgynna nfltppdgqa prmrmyiwdt atpyrdgdle agivi**QGYSA** glstrltggp

601 ansgclgyge aggmg**EGWGD** aiatlirqve ehknfengtd vfsmgawaan snrgirnyky

661 stnftinpst yktldkpgyw gvhaigevwa eflfvlsqrl vekygfgptl fpptdtskhn

721 dyytrtsees vdaagrplpl vpkygnalai qlivdamklq pcrpsffdar naiiqadqil

781 tggenacliw qafaerglge daavvgqtpw gggvrsdgfk vpkkvceskk a

***MPR1-QGAAA***

1 mrssaliall pflatltaar phhredkhsa srtrkslsfg pahshasfev lddavhvfep

61 rglidepidv krvaqtflgs qlgaqegegf yiredsytda rtgvthifar qllnglevsd

121 gdinlnidrd grimswgnsf hpgsvpslsd ihssssgete kvcttlhqhl dehkahlael

181 kgetgiwglv ksaaqvvlgs slplgevdhh eikethksmr hienhlramc dqpavstqsm

241 lspvealvsl lprlspiddl edispfdlts tphhtlkpkp afaepptevi sgaalskagv

301 vsdvsarlmy tqvsegaprl vwkyevemkd swyeayvdvl sgelirvvdw asdfdidelr

361 dkiemmkggk qkplpippkk iqpysyqvfp wgindpvsgn lsvvtepwdt vasplgwhsf

421 ptsanpwdvt ipgettnhny tvfnttagnn vyahenwegr nnfllnyrpt ndshifvyey

481 gepeglapke yvdmvvtqlf ytanmyhdll yrlgfdelsg nfqaynfrlg gkggdpvvcn

541 aqdgsgynna nfltppdgqa prmrmyiwdt atpyrdgdle agivi**QGYSA** glstrltggp

601 ansgclgyge aggmg**AGWGA** aiatlirqve ehknfengtd vfsmgawaan snrgirnyky

661 stnftinpst yktldkpgyw gvhaigevwa eflfvlsqrl vekygfgptl fpptdtskhn

721 dyytrtsees vdaagrplpl vpkygnalai qlivdamklq pcrpsffdar naiiqadqil

781 tggenacliw qafaerglge daavvgqtpw gggvrsdgfk vpkkvceskk a
