# Supplementary figures and images for: The structure-function analysis of the Mpr1 metalloprotease determinants of activity during migration of fungal cells across the blood-brain barrier
Source: PLoS One. 2018 Aug 30;13(8):e0203020. doi: 10.1371/journal.pone.0203020 (PMC6117016; doi:10.1371/journal.pone.0203020)

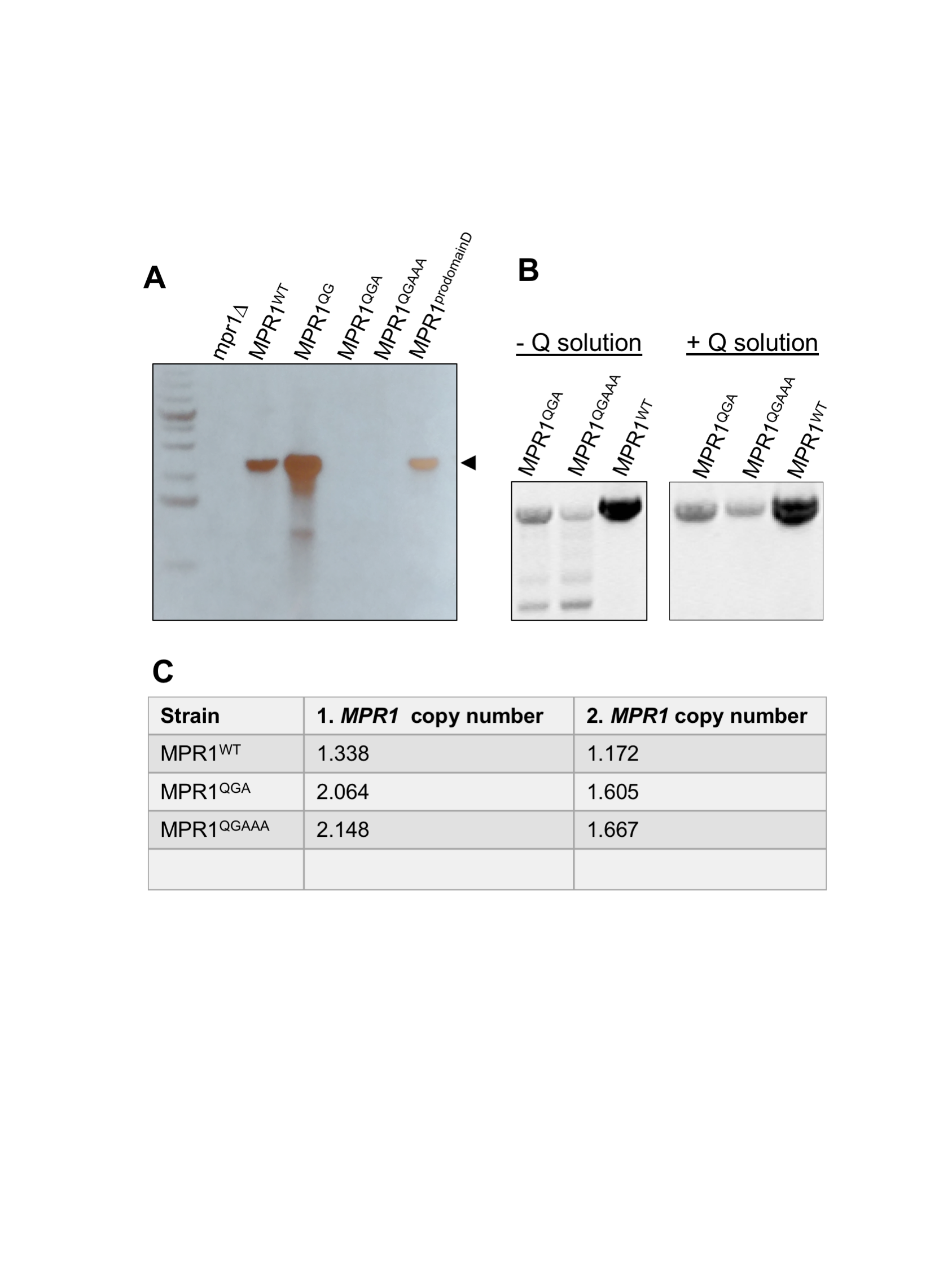

Supplement: S1 Fig — (A) Southern blot analysis detected a single MPR1 copy number in MPR1wt, MPR1QG and MPR1Prodomain transformed strains, suggesting mutant constructs were integrated as single copy. (B) Despite the negative Southern blot result for the remaining strains, the presence of MPR1 gene in MPR1QGA and MPR1QGAAA strains is confirmed by PCR. Q solution (Qiagen) is a PCR enhancing additive. (C) Real time PCR confirmed 1 to 2 copies of genomic MPR1 mutant in MPR1QGA and MPR1QGAAA strains. Real time PCR was performed with 2 different sets of primers. (TIFF) [file pone.0203020.s005.tiff]
